# A proposed framework for the development and qualitative evaluation of West Nile virus models and their application to local public health decision-making

**DOI:** 10.1371/journal.pntd.0009653

**Published:** 2021-09-09

**Authors:** Alexander C. Keyel, Morgan E. Gorris, Ilia Rochlin, Johnny A. Uelmen, Luis F. Chaves, Gabriel L. Hamer, Imelda K. Moise, Marta Shocket, A. Marm Kilpatrick, Nicholas B. DeFelice, Justin K. Davis, Eliza Little, Patrick Irwin, Andrew J. Tyre, Kelly Helm Smith, Chris L. Fredregill, Oliver Elison Timm, Karen M. Holcomb, Michael C. Wimberly, Matthew J. Ward, Christopher M. Barker, Charlotte G. Rhodes, Rebecca L. Smith

**Affiliations:** 1 Division of Infectious Diseases, Wadsworth Center, New York State Department of Health, Albany, New York, United States of America; 2 Department of Atmospheric and Environmental Sciences, University at Albany, Albany, New York, United States of America; 3 Information Systems and Modeling & Center for Nonlinear Studies, Los Alamos National Laboratory, Los Alamos, New Mexico, United States of America; 4 Center for Vector Biology, Rutgers University, New Brunswick, New Jersey, United States of America; 5 Department of Pathobiology, College of Veterinary Medicine, University of Illinois at Urbana-Champaign, Urbana, Illinois, United States of America; 6 Instituto Costarricense de Investigación y Enseñanza en Nutrición y Salud (INCIENSA), Tres Rios, Cartago, Costa Rica; 7 Department of Entomology, Texas A&M University, College Station, Texas, United States of America; 8 Department of Geography & Regional Studies, University of Miami, Coral Gables, Florida, United States of America; 9 Department of Ecology and Evolutionary Biology, University of California, Los Angeles, California, United States of America; 10 Department of Ecology and Evolutionary Biology, University of California, Santa Cruz, California, United States of America; 11 Department of Environmental Medicine and Public Health, Icahn School of Medicine at Mount Sinai, New York, New York, United States of America; 12 Institute for Exposomic Research, Icahn School of Medicine at Mount Sinai, New York, New York, United States of America; 13 Department of Environmental Health Sciences, Mailman School of Public Health, Columbia University, New York, New York, United States of America; 14 Department of Geography and Environmental Sustainability, University of Oklahoma, Norman, Oklahoma, United States of America; 15 Connecticut Agricultural Experimental Station, New Haven, Connecticut, United States of America; 16 Northwest Mosquito Abatement District, Wheeling, Illinois, United States of America; 17 Department of Entomology, University of Wisconsin-Madison, Madison, Wisconsin, United States of America; 18 School of Natural Resources, University of Nebraska-Lincoln, Lincoln, Nebraska, United States of America; 19 National Drought Mitigation Center, University of Nebraska-Lincoln, Lincoln, Nebraska, United States of America; 20 Mosquito and Vector Control Division, Harris County Public Health, Houston, Texas, United States of America; 21 Department of Pathology, Microbiology, and Immunology, University of California Davis, California, United States of America; 22 Environmental Analytics Group, Universities Space Research Association, NASA Ames Research Center, Moffett Field, California, United States of America; 23 Department of Tropical Medicine, Tulane University School of Public Health & Tropical Medicine, New Orleans, Louisiana, United States of America; Australian Red Cross Lifelood, AUSTRALIA

## Abstract

West Nile virus (WNV) is a globally distributed mosquito-borne virus of great public health concern. The number of WNV human cases and mosquito infection patterns vary in space and time. Many statistical models have been developed to understand and predict WNV geographic and temporal dynamics. However, these modeling efforts have been disjointed with little model comparison and inconsistent validation. In this paper, we describe a framework to unify and standardize WNV modeling efforts nationwide. WNV risk, detection, or warning models for this review were solicited from active research groups working in different regions of the United States. A total of 13 models were selected and described. The spatial and temporal scales of each model were compared to guide the timing and the locations for mosquito and virus surveillance, to support mosquito vector control decisions, and to assist in conducting public health outreach campaigns at multiple scales of decision-making. Our overarching goal is to bridge the existing gap between model development, which is usually conducted as an academic exercise, and practical model applications, which occur at state, tribal, local, or territorial public health and mosquito control agency levels. The proposed model assessment and comparison framework helps clarify the value of individual models for decision-making and identifies the appropriate temporal and spatial scope of each model. This qualitative evaluation clearly identifies gaps in linking models to applied decisions and sets the stage for a quantitative comparison of models. Specifically, whereas many coarse-grained models (county resolution or greater) have been developed, the greatest need is for fine-grained, short-term planning models (m–km, days–weeks) that remain scarce. We further recommend quantifying the value of information for each decision to identify decisions that would benefit most from model input.

## Introduction

West Nile virus (WNV) is one of the most widely distributed mosquito-borne viruses and represents a global public health threat [1,2]. In the United States, WNV is the most common vector-borne virus with at least 51,801 human cases and 2,390 fatalities reported between its introduction in 1999 and 2019 [3]. WNV has had substantial negative economic impacts through healthcare costs (about $368 million to $2.4 billion in Texas in 2012) [4] and in equine-related veterinary financial burdens (e.g., $1.9 million in 2002 in North Dakota prior to the vaccine in 2004) [5]. In addition to human and veterinary disease, WNV has impacted avian populations being reported in over 300 species of birds in the US [6]. The virus killed millions of songbirds [7] and led to population declines in some species, in particular American crows (*Corvus brachyrhynchos*) [8,9], ruffed grouse (*Bonasa umbellus*) [10], and yellow-billed magpies (*Pica nuttalli*) [9,11]. Effective preparedness and prevention are vital to reduce the direct and indirect impacts of WNV on human health, the environment, and the economy.

The majority of human cases of WNV are asymptomatic (approximately 80%) [12]. Symptomatic cases are classified as either non-neuroinvasive or as neuroinvasive, with neuroinvasive cases representing <1% of cases [12,13]. Many non-neuroinvasive cases of WNV go unreported. However, due to the severity of symptoms, neuroinvasive case reports are expected to be less biased [14]. Mosquito infection rates are typically expressed as either minimum infection rates (MIRs) or maximum likelihood estimates of infection rate (MLE). MIR is in common usage; however, MLE is more accurate and conveys more information [15,16].

Numerous statistical [17] and mathematical [18] models have been developed to understand and predict WNV geographic and temporal dynamics that could be potentially used to guide vector control and public health activities. Models can be used to understand the relationships between the spatial distribution of human pathogens, vectors, the prevalence of vector-borne diseases, including social demographic and environmental predictors [19]. Notably, the model requirements and desired end result may vary depending on the decision-making mechanisms of stakeholders, and it is unlikely that there will be a single model that is suitable for all decisions. A recent review classified 48 WNV models as risk, detection, or warning models [17]. Risk models provide spatial information about relative risk, but do not contain temporal information. Detection models make estimates for the current season but do not integrate the current year’s WNV surveillance data. Early warning models include current-year WNV surveillance data. Most models make predictions at a single broad or narrow spatial or temporal scale, whereas decisions occur at multiple scales. Decisions on where and when to apply larvicide (to control immature aquatic stages) or adulticide (to control vagile adults) are usually made on a weekly timescale and a relatively fine spatial scale such as city blocks or neighborhoods. In contrast, decisions on hiring and staffing for control need to be made on a monthly or seasonal timescale at the level of the local mosquito control agency and often in advance of a transmission season.

In this review, we developed a framework for applying models to decisions and tested it on 13 representative models that have been developed to understand and predict WNV geographic and temporal dynamics. These models include descriptive statistical models, a mathematical mechanistic model, and data assimilation–based models. Specifically, we asked the question “what information does each model provide about WNV?” It is critical that state, tribal, local, or territorial public health and mosquito control agencies understand WNV model outputs and find them useful in operational support. We compared model properties, inputs, and outputs in the context of these public health decisions. Specifically, we examined the capacity and suitability of the models with respect to spatial and temporal scales to guide the timing and the locations for mosquito and virus surveillance, to support mosquito vector control decisions, and to assist in conducting public health outreach campaigns.

## Materials and methods

**Fig 1:**
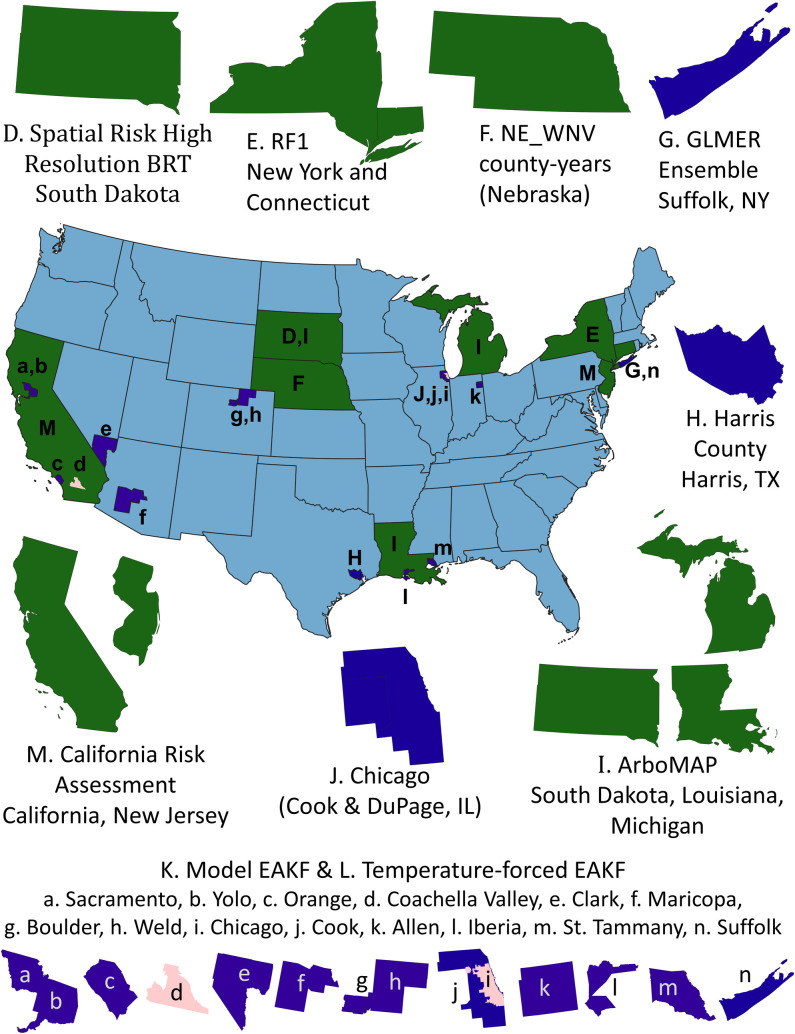
Map of specific locations where WNV models included in this comparison have been applied. Some models (Spatial Risk Random Forest, not shown) have been applied across the entire US. Green corresponds to analyses with state extents, blue to county extents, and pink to subcounty extents. State outlines are from Natural Earth (https://www.naturalearthdata.com/downloads/50m-cultural-vectors/). City of Chicago boundary is publicly available from the City of Chicago (https://data.cityofchicago.org/Facilities-Geographic-Boundaries/Boundaries-City/ewy2-6yfk), and county boundaries and the outline for Coachella Valley were derived from US Census tract boundaries (https://www.census.gov/geographies/mapping-files/time-series/geo/carto-boundary-file.html) dissolved to provide a single outline using the Dissolve algorithm in QGIS (https://qgis.org/en/site/). WNV, West Nile virus.

We aimed to review models that were in active development or currently being applied to the West Nile virus system by decision-makers. Rather than identify individual models, we identified research teams studying spatiotemporal dynamics of WNV based on academic conference presentations, recent publications, involvement in one of the 5 Centers for Disease Control and Prevention (CDC) Regional Centers of Excellence, and referral to participate in a workshop hosted by the National Socio-Ecological Synthesis Center (SESYNC). We aimed to identify and include a diverse audience of participants to minimize various biases. We were initially capped at 25 participants but expanded the West Nile Virus Model Comparison Project participant list to 35 when the workshop was moved to a virtual format. With respect to primary affiliation, 74.3% were from academia, 11.4% from a department of health, and 14.3% in vector control. The 13 models included in this review cover much of the regional variation within the US (Fig 1).

By taking a research team–based approach, we were able to take an in-depth look at each of the selected models. This in-depth analysis would not have been possible with in a traditional review format. The models selected here are broadly representative of the WNV models that have been developed (e.g., statistical [20–24], data assimilation [25,26], mathematical trait–based [27], machine learning [28–30], threshold-based risk [31–33], and distributed lag approaches [34–36]). We also include a probabilistic historical null model in our comparison [37]. Our framework is reproducible providing the templates for model description (S1 Text) and instructions (S2 Text), the template questions for decision-makers (S3 Text), and detailed descriptions of each model (S4 Text), so any omitted or future models could be evaluated and compared following the framework outlined here.

**Fig 2:**
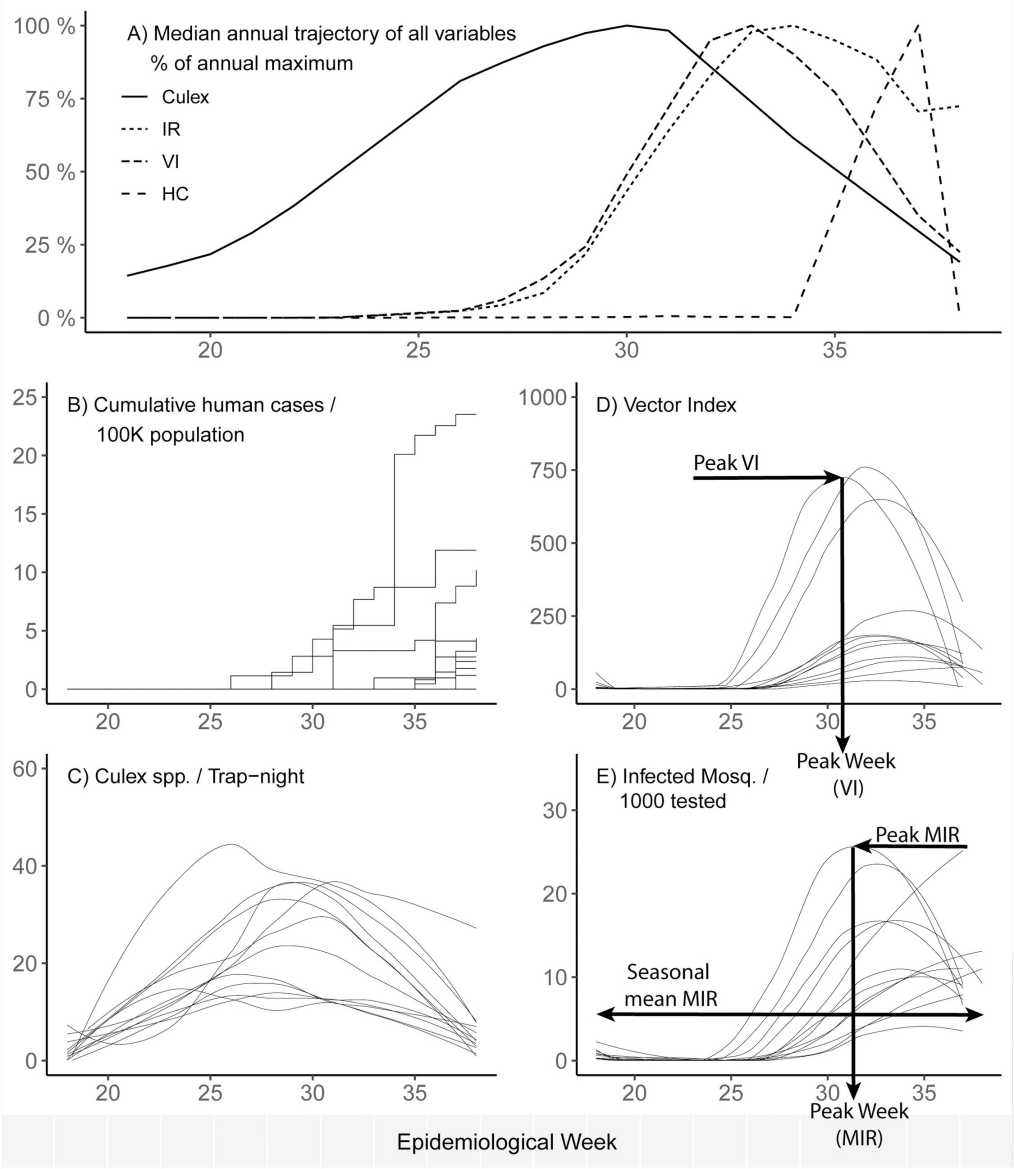
Examples of key model outputs. (A) A summary of key outputs for 1 year. (B) Cumulative human cases (annual human cases), (C) *Culex* mosquito abundance per trap night, (D) vector index (*Culex* abundance times infection rate by week), and (E) MIR per 1,000 mosquitoes. Peak MLE/IR is the mosquito infection rate in the peak week, Peak week for MLE/IR is the week in which the peak is reached, while Seasonal MLE/MIR is the infection rate over the season when the mosquitoes are active (using either MLEs or MIRs). Culex, *Culex* abundance; IR, mosquito infection rate, either as MIR or MLE; HC, human cases; MIR, minimum infection rate; MLE, maximum likelihood estimate of infection rate; VI, vector index.

**Fig 3:**
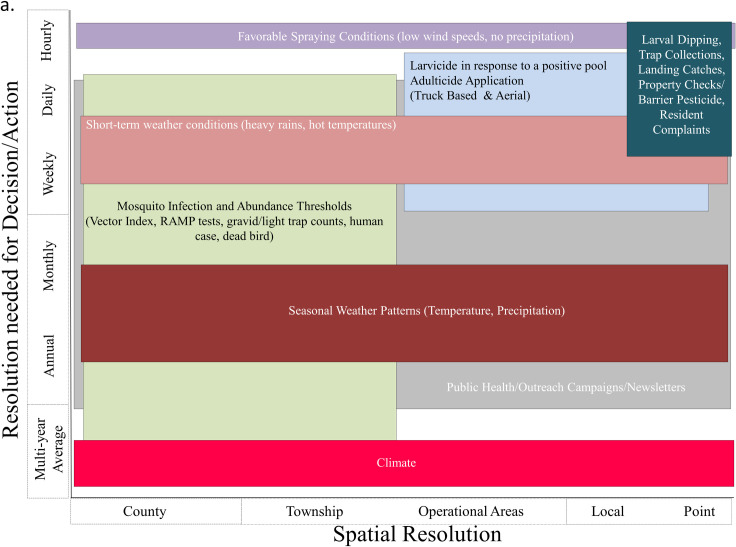
Generalized overview of major factors, tools, and decisions utilized by mosquito control agencies. This figure is based on 4 representative mosquito abatement districts: 2 in Chicago (IL), Slidell (LA), and Houston (TX). Management practices may differ from program to program, but similar challenges and decisions are made from across varying spatial (local to district-wide) and temporal (days to multiple months) scales.

**Fig 4:**
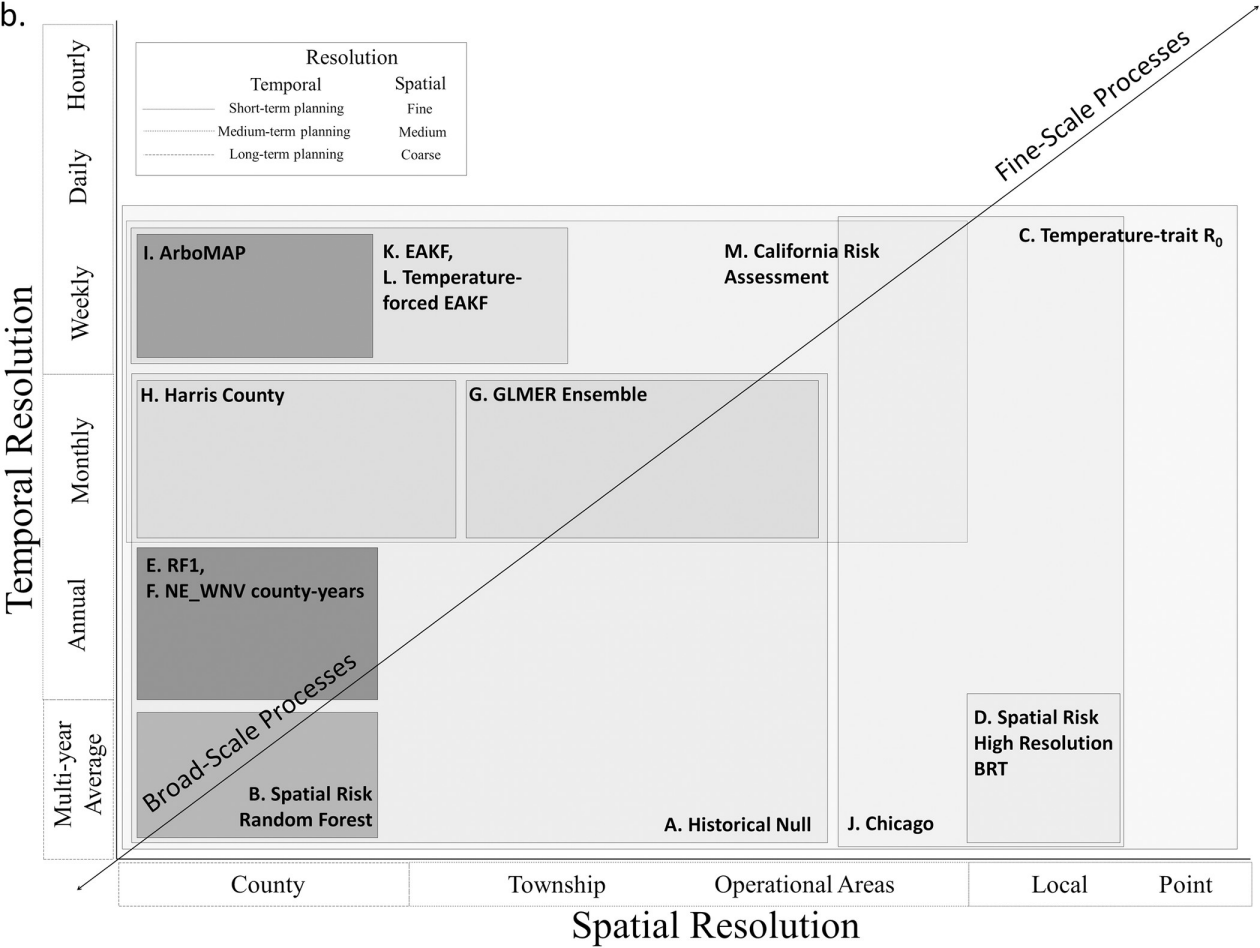
The 13 models reviewed in this paper arranged by spatial and temporal resolution. Rectangles with decreasing shades of gray indicate less coverage identifying potential knowledge gaps. These gaps may guide future model development or require additional data collection, as many models are at the county-annual scale due to data availability.

**Fig 5:**
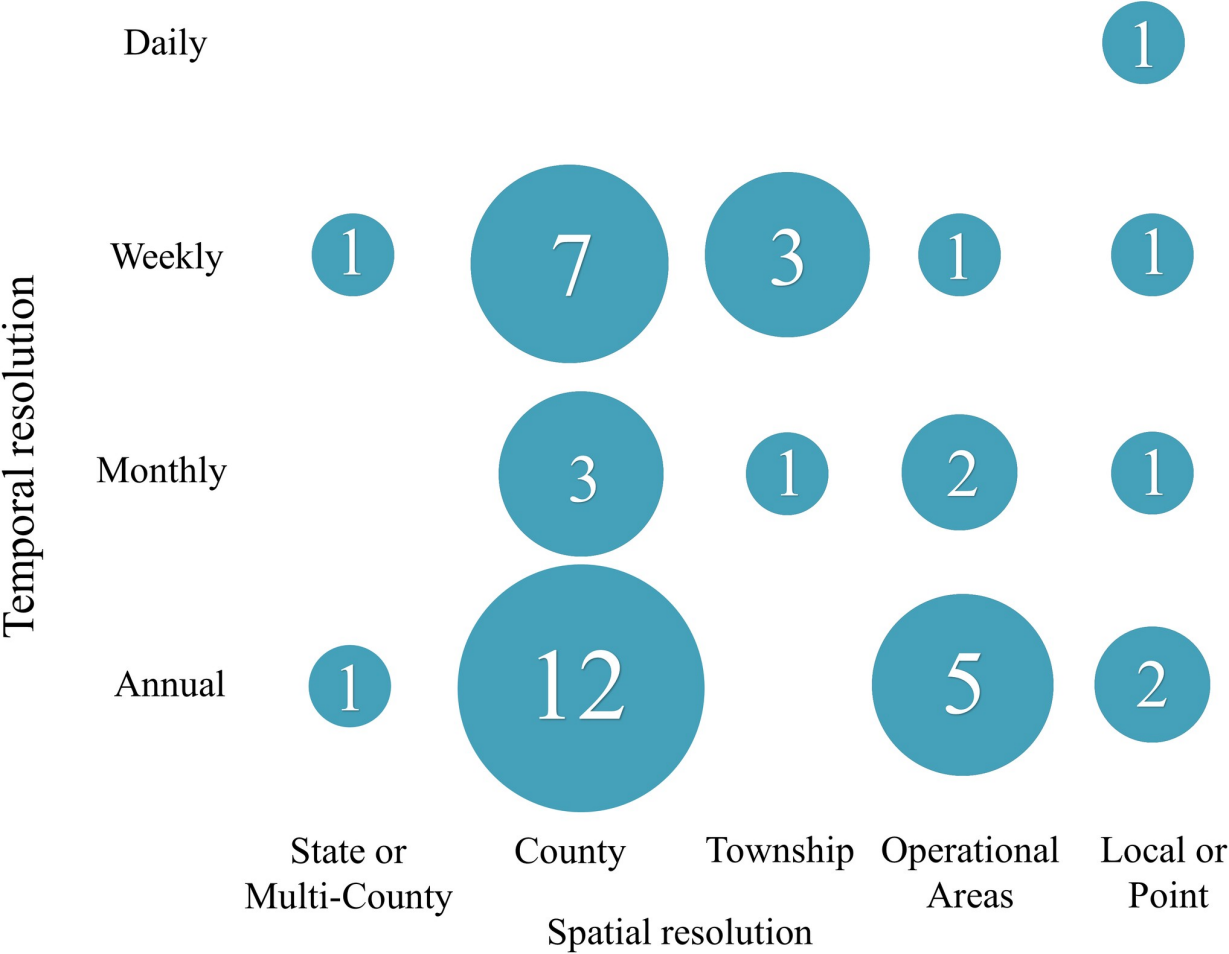
A summary of the spatial and temporal resolution for the 41 models reviewed in [17] that are not included in Fig 4. Numbers indicate the number of models at that spatial and temporal scale.

This paper is organized starting with an overview of all models (Table 1), model inputs (Table 2), model outputs and predictions (Fig 2 and Table 3), and, finally, model applications (Table 4). Decisions and models are compared with respect to temporal and spatial resolution (Figs 3–5). The model description template used to collect model information is available as S1 Text, and the detailed description of all fields is provided in S2 Text. This framework lays the foundation for qualitative assessment as a precursor to more rigorous quantitative comparisons for those models that are suitable for the intended purposes. The majority of the models have been published; for those models, additional details can be found in the respective publications (see citations in Table 4). Computation time will depend on the computer used, and no formal benchmarking was performed for the models. However, all models except the GLMER Ensemble are expected to run in under an hour for a county or smaller extent on a PC with a 2.9 GHz processor and 16 GB RAM.

**Table 1:** Model overview: A comparison of model class, spatial, temporal resolution, software implementation, and code availability.

Model	Class of Model^1^	Spatial Resolution	Temporal Resolution	Software	Code Available
A. Historical Null	Spatial patterns	Flexible	Annual^2^	R	www.github.com/akeyel/dfmip
B. Spatial Risk Random Forest	Spatial patterns	County	Mean from 2005–2018	R	No
C. Temperature-trait-based Relative R_0_ Model	Spatial patterns	Flexible	Flexible	R	https://datadryad.org/stash/dataset/doi:10.5068/D1VW96
D. Spatial Risk High Resolution BRT Model	Spatial patterns	300 × 300 m	Mean from (2004–2017)	R	No (in progress)
E. RF1	Early warning	Flexible	Annual	R	www.github.com/akeyel/rf1
F. NE_WNV County-years	Early warning	County	Annual	R, mgcv	www.github.com/khelmsmith/flm_NE_WNV
G. GLMER Ensemble	Early warning	13 × 13 km grid	Monthly	R	No
H. Harris County	Early warning Early detection	Whole Harris County	Month	R	Based on code for SAR models presented by [38]
I. ArboMAP	Early detection	Typically county	Weekly	R	https://github.com/EcoGRAPH/ArboMAP/releases/
J. Chicago Ultra-Fine Scale	Early detection	1-km hexagon	1 week (epi weeks 18–38)	JMP, SAS	No (in progress)
K. Model-EAKF System	Early detection	Mosquito abatement district	Weekly	Matlab/R	Available upon request
L. Temperature-forced Model-EAKF System	Early detection	Mosquito abatement district	Weekly	Matlab/R	Available upon request
M. California Risk Assessment	Early detection	Flexible	Flexible	VectorSurv Gateway (website)^3^	Available upon request

^1^Spatial patterns: models with predictions that do not vary by year. Early warning: models that do not include current-year surveillance data, may include current-year climate/weather data, and have a model lead time on the order of days to months. Early detection: models that include current-year surveillance data, may include other data streams, and have a lead time on the order of days to months.

^2^The model itself is flexible with respect to temporal resolution. The GitHub implementation was designed for annual temporal resolution.

^3^The website is implemented in Javascript, PHP, SQL, Google Maps API, and Mapbox API.

**Table 2:** Model inputs.

Model	Human Data	Mosquito Surveillance	Climate/Weather	Land-cover	Sociological	Other
A. Historical Null	Y^1^	Y^1^	N	N	N	N
B. Spatial Risk Random Forest	Y	N	Y	N	N	N
C. Temperature-trait-based Relative R_0_ Model	N	N	Y	N	N	N
D. Spatial Risk High Resolution BRT	Y	N	Y	Y	N	Y
E. RF1	Y	Y	Y	Y	Y	Y
F. NE_WNV County-years	Y	N	Y	N	N	N
G. GLMER Ensemble	N	Y	Y	N	N	N
H. Harris County	N	Y	Y	Y	N	Y
I. ArboMAP	Y	Y	Y	N	N	N
J. Chicago Ultra-Fine Scale	Y	Y	Y	Y	Y	Y
K. Model-EAKF System	Y	Y	N	N	N	Y
L. Temperature-forced Model-EAKF System	Y	Y	Y	N	N	Y
M. California Risk Assessment	Y	Y	Y	Y	N	Y

^1^For the Null model, only human data are required to predict human cases, and only mosquito surveillance data are required to predict mosquito infection rates. Mosquito surveillance is not used to predict human cases or vice versa in this model.

**Table 3:** Model output/predictions. Prediction targets included human case counts, mosquito infection rates as either MIRs or as MLEs. Probabilistic models are those that generate predictions as probability distributions rather than single mean values. The additional prediction targets column indicates whether the model generates additional outputs not otherwise included in the table.

Model	Annual Human Cases	Seasonal MLE/MIR	Peak MLE/MIR	Peak Week for MLE/MIR^1^	Vector Index (weekly)	Probabil-istic?	Additional Prediction Targets
A. Historical Null	Y	Y	N	N	N	Y	N
B. Spatial Risk Random Forest	N	N	N	N	N	N	Y
C. Temperature-trait-based Relative R_0_ Model	N	N	N	N	N	Y	Y
D. Spatial Risk High Resolution BRT	N	N	N	N	N	Y	Y
E. RF1	Y	Y	N	N	N	Y^2^	N
F. NE_WNV County-years	Y	N	N	N	N	Y	Y^3^
G. GLMER Ensemble	N	Y	N	N	N	N	N
H. Harris County	N	Y	Y	Y	N	N^4^	Y
I. ArboMAP	Y	Y	N	N	N	Y	Y
J. Chicago Ultra-Fine Scale	Y	N	N	N	N^5^	Y	Y^6^
K. Model-EAKF System	Y	Y	Y	Y	N^7^	Y	Y^8^
L. Temperature-forced Model-EAKF System	Y	Y	Y	Y	N^7^	Y	Y^8^
M. California Risk Assessment	N	N	N	N	N	N	Y^9^

^1^Peak week could also be calculated for human cases but typically is not done in practice; therefore, this output was omitted from the table.

^2^The model has been upgraded since the initial publication to support probabilistic outputs.

^3^Counties with cases.

^4^In principle, the model could produce probabilistic output.

^5^The model uses vector index as a predictor but does not predict values for vector index.

^6^Can theoretically inverse cases and MIR, but model not tested for that.

^7^The model can be parameterized with either MLE infection rates or vector index, but empirically, the results from the vector index parameterization were not as strong, and, therefore, the final model is based on MLE.

^8^+/−25% of peak week, human cases, total infections over the season; +/−25% or 1 human case.

^9^Virus transmission risk to humans.

BRT, Boosted Regression Trees; EAKF, Ensemble-adjustment Kalman Filter; MIR, minimum infection rate; MLE, maximum likelihood estimate of infection rate.

**Table 4:** Model applications. Only published model applications were included. Each line corresponds to a separate model test; therefore, some models appear more than once. References are listed for further details.

Model	Study	Prediction Target	Sample Size	Spatial Domain	Time Domain	Testing Method^1^	Metric Score^2^
B. Spatial Risk Random Forest	[29]	Mean annual incidence per 100,000 population	43,512 county-years	Conterminous US (3,108 counties)	2005–2018, averaged	Bootstrapping	*R*^*2*^_*pred*_ = 0.59 [0.44–0.70], *RMSE* = 3.7
D. Spatial Risk High Resolution BRT	[30]	Ranked relative risk (0–1)	1,378 human cases	South Dakota	2004–2017	Out of sample data	*AUC* = 0.727
E. RF1	[28]	Annual human cases	882 county-years	New York and Connecticut	2000–2015	LOYOCV	*R*^*2*^_*pred*_ = 0.72, *RMSE* = 1.6
E. RF1	[28]	Seasonal mosquito MLE	218 county-years	New York and Connecticut	2000–2015	LOYOCV	*R*^*2*^_*pred*_ = 0.45, *RMSE* = 2.3
E. RF1	[28]	Seasonal mosquito MLE	2,596 trap-years	New York and Connecticut by trap	2000–2015	LOYOCV	*R*^*2*^_*pred*_ = 0.53, *RMSE* = 1.0
F. NE_WNV County-years	[34]	2018 human cases^3^	1,472 county-years	Nebraska	2002–2017	Out of sample data	*CRPS* = 1.90
F. NE_WNV County-years	[34]	2018 WNV positive counties^3^	1,472 county-years	Nebraska	2002–2017	Out of sample data	*Accuracy* = 0.717
G. GLMER Ensemble	[20]	MLE mosquito infection rate	225 grid-years	Suffolk County, New York	2001–2015	LOYOCV	*RMSE* = 4.27
H. Harris County	[21]	MLE mosquito infection rate (1-month lead)	130,567 trap-nights	Harris County, Texas	2002–2016	Out of sample data	*R*^*2*^_*pred*_ = 0.8
H. Harris County	[21]	Mosquito abundance (1-month lead)	10,533,033 mosquitoes	Harris County, Texas	2002–2016	Out of sample data	*R*^*2*^_*pred*_ = 0.2
I. ArboMAP	[35]	Positive county-weeks	Approximately 9,504 county-weeks (training) Approximately 792 county-weeks (testing)	South Dakota	2004–2015 (training) 2016 (testing)	Out of sample data	*AUC* = 0.836–0.856^4^
I. ArboMAP	[36]	Positive county-weeks	Approximately 11,088 county-weeks	South Dakota	2004–2017^5^	Fit to training data only	*AUC* = 0.876, *R*_*s*_ = 0.84
J. Chicago Ultra-Fine Scale	[22–24]	Human case probability (by hexagon)	1,346,940 hexagon-weeks	Variable, up to 5,345 1-km hexagons	2005–2016^6^	Fit to training data only	*R*^*2*^ > 0.85; *RMSE* < 0.02; *AUC* > 0.90
K. Model-EAKF System	[25]	Annual human cases; peak mosquito infection rates; peak timing of infectious mosquitoes; annual infectious mosquitoes	21 county-years	2 counties (Suffolk, New York and Cook, Illinois)	Weekly, Varied by location	Retrospective data assimilation	*Threshold-based accuracy* ^7^
K. Model-EAKF System	[26]	Multiple^8^	110 outbreak-years	12 counties	Weekly, Varied by location	Retrospective data assimilation	*Threshold-based accuracy* ^7^
K. Model-EAKF System	[39]	Multiple^8^	4 county-years	4 counties	Weekly, 2017	Real-time data assimilation	*Threshold-based accuracy* ^7^
L. Temperature-forced Model-EAKF System	[26]	Multiple^8^	110 outbreak- years	12 counties	Weekly, Varied by location	Retrospective data assimilation	*Threshold-based accuracy* ^7^
M. California Risk Assessment	[31]	Historical outbreaks of western equine encephalomyelitis and St. Louis encephalitis as proxy for WNV	14 agency-years	California	Half-months	Temporal correspondence	Early detection of arbovirus risk prior to outbreaks
M. California Risk Assessment	[32]	Onset and peak of human cases by geographic region	12 half-months in 3 regions	California	Half-months	Retrospective data assimilation	Early detection of WNV risk prior to onset and peak of human cases
M. California Risk Assessment	[33]	Emergency planning threshold (risk ≥ 2.6)	11,476 trap-nights	Los Angeles Country, California	2004–2010	Retrospective data assimilation	*AUC* = 0.982

^1^LOYOCV: leave-one-year-out cross-validation; Out of sample data: accuracy based on data not used to develop the model; Fit to training data only: accuracy based on the same data used to develop the model; Retrospective data assimilation: finalized data until the time of forecast; Real-time data assimilation: data processed and available at the time of forecast.

^2^*R*^*2*^_*pred*_: predictive R^2^, i.e., an R^2^ calculated on data outside the sample, *R*_*s*_: Spearman correlation coefficient, *AUC*: area under the curve, *Threshold-based accuracy*: +/−25% of peak week, human cases, total infections over the season; +/−25% or 1 human case, *RMSE*: Root Mean Squared Error, *CRPS*: Continuous Ranked Probability Score.

^3^Results for 2018 reported here, validation was also performed separately for 2012–2017, see [34] for details.

^4^Three analyses presented: short-term: AUC = 0.856, annual made on July 5: AUC = 0.836, annual made on July 39: AUC = 0.855.

^5^Restricted to July–September for each year.

^6^Restricted to 21 epi weeks per year.

^7^Varied by analysis and lead time.

^8^Prediction targets: human cases in next 3 weeks; annual human cases; week with highest percentage of infectious mosquitoes; peak mosquito infection rate; annual infectious mosquitoes.

AUC, area under the curve; CRPS, Continuous Ranked Probability Score; LOYOCV: leave-one-year-out cross-validation; RMSE, Root Mean Squared Error; WNV, West Nile virus.

In order to understand public decision-making processes and goals, we sent a request for information on what decisions are routinely made with respect to WNV (Table 5, form provided as S3 Text), through the Centers for Disease Control Regional Centers of Excellence. We received responses from 4 mosquito abatement districts (St. Tammany Parish Mosquito Abatement, LA; Northwest Mosquito Abatement District, IL; North Shore Mosquito Abatement District, IL; and the Harris County Mosquito and Vector Control Division, TX). We categorized the responses and then compared the suitability of each model with respect to temporal and spatial scale in relation to each decision identified by the public health professionals, until a consensus was reached.

**Table 5:** List of common decisions made regarding a public health and vector control response to WNV. Letters correspond to models in Tables 1–4 and indicate models with an appropriate spatial or temporal resolution to inform the decision. Note that this pertains to the scale on which predictions are made and provides no information on the accuracy of the model predictions. As such, models with appropriate scale, but insufficient accuracy, would not be useful in an operational context.

Public health decisions	Potentially applicable models	
	When (timing)	Where (area)
Mosquito and WNV surveillance (trap sites)	**C, M**	**C, D, M**
Mosquito and WNV surveillance (county/district thresholds)	**C, M**	**A–J, M**
Public health and outreach	**C, E–M**	**A–J, M**
Larviciding	**C, H–M**	**C, J, M**
Truck-based adulticiding	**C, I–M**	**C, J, M**
Aerial adulticiding	**C, I–M**	**C, J, M**

WNV, West Nile virus.

### Model classification framework

**Fig 6:**
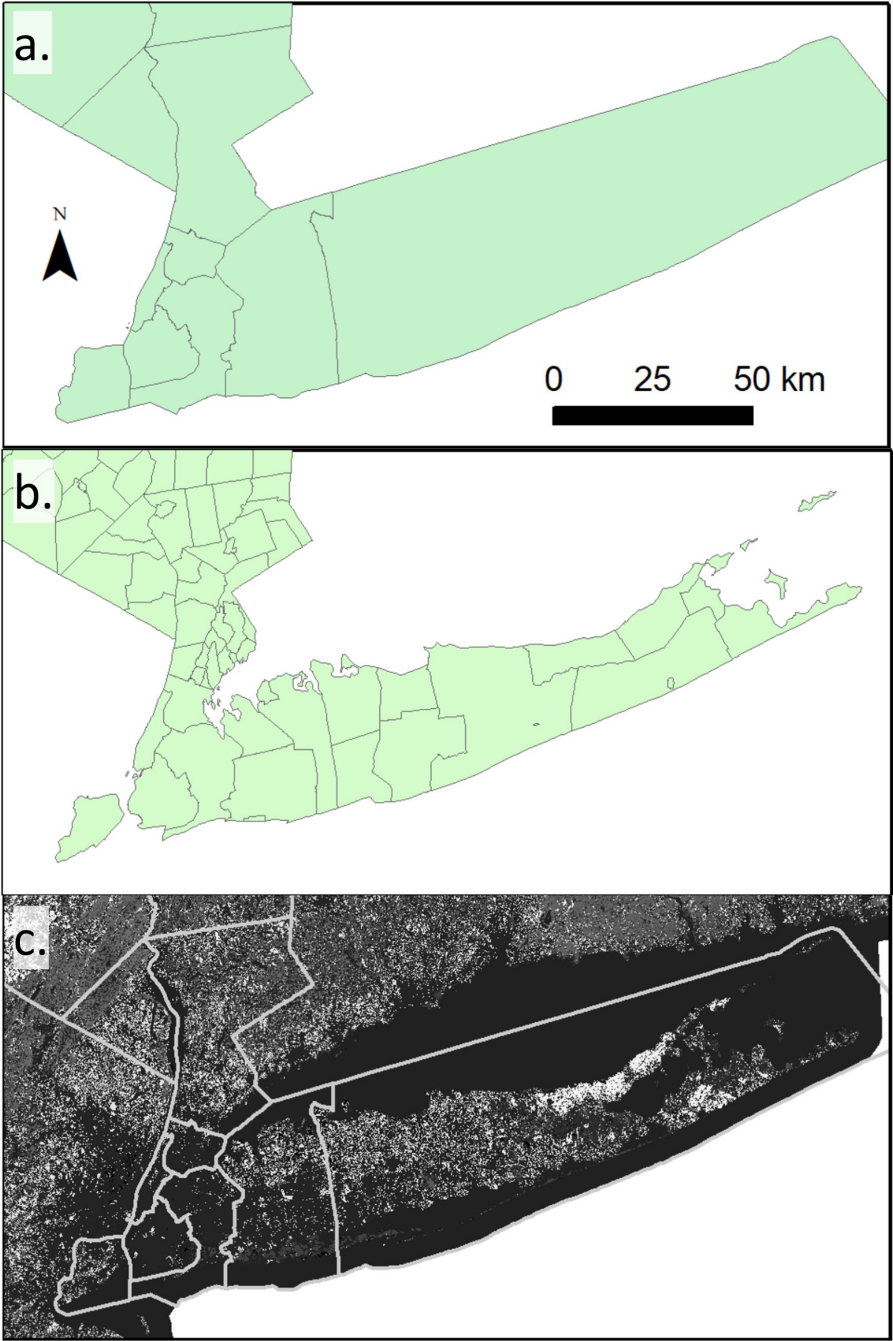
Examples of the 3 spatial scales described in Table 6 for Long Island, NY. (a) Coarse-grain: county, (b) medium-grain: county subdivision, and (c) fine-grain: 30 × 30 m resolution for vegetation types [40], with the NY county outlines in gray for context. County outlines and county subdivisions from the 2017 US Census https://www.census.gov/geo/maps-data/data/tiger-line.html).

The models varied in spatial and temporal resolution. Based on the distribution of temporal and spatial resolutions in Fig 4, we propose a 3 × 3 description system for models based on their temporal and spatial scales (Table 6). We define 3 temporal scales: (1) short-term, corresponding to operational decisions made on the scale of weeks to months; (2) medium-term, corresponding to decisions made over months within a year related to planning and preparation; and (3) long-term for planning efforts made across multiple years. With respect to spatial scale, we identify fine-grain models with resolution of meters to kilometers (e.g., Fig 6C), medium-grain models with a resolution of a single management unit (e.g., mosquito abatement districts or county subdivisions; Fig 6B), and coarse-grain models that make predictions for multiple aggregated management units or single management units with large geographic coverage (e.g., county-level models; Fig 6A). Note that these scales apply to the resolution of the models, not the extent of the models. For example, weekly risk estimates with a 30 m × 30 m cell size would be a short-term fine-grain model, regardless of whether it was applied to a single neighborhood or an entire country. The aim of this description system is to better align model descriptions with the scales of application. For example, decisions requiring a short-term fine-grain model, such as where to apply an adulticide, would not be informed by a medium-term coarse-grain planning model. We suggest that models could also be classified based on lead time and accuracy, sensitivity, and specificity, but these classifications may be region and/or scale dependent and, therefore, require a rigorous quantitative comparison to be developed.

**Table 6:** Classification of temporal and spatial resolutions relevant to vector control and public health decision-making.

Classification Term	Spatial or Temporal	Resolution
Long-term planning	Temporal	Years to decades
Medium-term planning	Temporal	Months to year
Short-term planning	Temporal	Days to weeks
Coarse grain	Spatial	Multiple/large management districts (e.g., county or above)
Medium grain	Spatial	Single management district or county subdivision
Fine grain	Spatial	Meters to km, within a management district

### Overview of models

The individual models are described in detail in S4 Text. Here, we summarize the models with respect to the purpose for which they were developed, the statistical basis of each model, the models’ use of surveillance data and climate data, and, finally, on their model selection approaches.

#### Model purposes

The models included in this review were developed for a variety of purposes, including generating present-day patterns of spatial risk, predicting risk under future climate change, and providing medium- to short-term planning guidance (see Table 6). The Spatial Risk High Resolution BRT model [30], Spatial Risk Random Forest model [29], and Temperature-trait-based Relative R_0_ models [27] were all developed for spatial risk, with the latter two with an aim to also provide information about climate change risk. The RF1 model can also been used to make climate change risk predictions [87].

Medium-term risk guidance models include the RF1 model and the NE_WNV County-years model [34] for human cases at the county-annual scale (and mosquito-based risk for RF1), and the GLMER Ensemble [28] and the Harris County models aimed at mosquito-based infection rates (and abundance for the Harris County model) at the monthly scale. ArboMAP (Arbovirus Modeling and Prediction) [35,36], the Model-EAKF Systems [25,26], the California Risk Assessment [31–33], and the Chicago Ultra-Fine Scale (UFS) models [23,24] all provide short-term risk information. Model-EAKF System and the Chicago UFS models model human cases directly. ArboMAP focuses on the probability of a county having at least 1 human case in a given week, while the California Risk Assessment model provides an index of relative risk without a quantitative prediction of numbers of human cases. ArboMAP was specifically designed to facilitate WNV forecasting by epidemiologists working in state public health offices, while the California Risk Assessment model is currently used by the state of California to guide vector control operations [41]. The Model-EAKF Systems provide a data assimilation approach, which uses data from the current season to update the model predictions as the season progresses to make weekly predictions in areas with high levels of mosquito surveillance.

#### Statistical basis

Broadly, the models fall into 3 general approaches: machine learning techniques, traditional statistical approaches, and mathematical models. For the machine learning approaches, Hess and colleagues used Boosted Regression Trees [42], while the Spatial Risk Random Forest Model and the RF1 Models used Random Forest methods [43,44]. The RF1 Model was modified to produce probabilistic output using quantile random forests [45,46].

Traditional statistical approaches include the GLMER Ensemble, using negative binomial mixed-effects models (GLMER Ensemble). The Harris County, TX Model is a seasonally autoregressive forced model [21], i.e., a linear model that capture nonsymmetric features in the seasonality of the underlying data. The NE_WNV County-years model [34] used a general additive model with thin-plate splines (the R package mgcv) [47] for nonparametric modeling of distributed lags (lag lengths of 12, 18, 24, 30, and 36 months) of drought and temperature data, using restricted maximum likelihood estimation with a log link and negative binomial distribution. The ArboMAP model also used a distributed lags approach. ArboMAP used logistic regression models with environmental indices (temperature, precipitation, humidity, etc.) included as distributed lags, with shapes governed by splines [35,36]. The Chicago UFS model is also based on logistic regression, with 1 km-wide hexagonal (spatial) and 1-week (temporal) resolutions using environmental, land-use/land-cover, historical weather, light pollution, human socioeconomic and demographic, mosquito abundance and infection, mosquito landing rates on humans, and human activity/exposure risk as covariates.

For the mathematical models, the Temperature-trait-based Relative R_0_ model used a modified Ross–McDonald equation that incorporates nonlinear thermal response curves fit to laboratory mosquito and virus trait data. The Model-EAKF System [25] and Temperature-forced Model-EAKF System [26] used a standard susceptible–infected–recovered epidemiological construct and were optimized using a data assimilation method (the ensemble adjustment Kalman filter (EAKF) [48] and 2 observed data streams: mosquito infection rates and reported human WNV cases. The models differ in that the Temperature-forced Model-EAKF System accounted for temperature modulation of the extrinsic incubation period for mosquitos [26]. The California Risk Assessment model estimates an overall level of WNV risk based on the average of all available risk elements (1) average daily temperature; (2) relative abundance of adult *Culex* mosquitoes versus the historical average; (3) WNV infection prevalence in *Culex* mosquitoes; (4) sentinel chicken seroconversions; (5) WNV infections in dead birds; and (6) human cases. Because human cases are affected by reporting lags and thus are unreliable indicators of real-time risk, they are typically omitted from risk calculations that guide mosquito control operations during the season. Each surveillance element is assigned a value on an ordinal scale (1 to 5 for lowest to highest risk), and the mean value of all factors is calculated to estimate the WNV transmission risk and corresponding response level (i.e., normal season (1.0 to 2.5), emergency planning (2.6 to 4.0), and epidemic (4.1 to 5.0)).

#### Use of surveillance data

Models varied in their use of mosquito and human surveillance data. Models using mosquito data calculated infection rates but differed in the approaches used to do so. The RF1 model used a published R method [49] applied at the county level, pooled for 3 *Culex* species: *Culex pipiens*, *Culex restuans*, and *Culex salinarius*. The Harris County Model used the maximum likelihood method by Farrington [50]. In the GLMER Ensemble, the *Culex* spp. infection rate was calculated for each NLDAS grid cell and year using maximum likelihood approaches. Similarly, the 2 Model-EAKF Systems estimate mosquito infection rates by week but require at least 1 positive mosquito and at least 300 mosquito samples per week. In ArboMAP, mosquito data are modeled in their own mixed-effects models, in which exponential growth curves are imposed on mosquito infection rates in the early season. The estimated growth rate is then used as a covariate in the human models. The Chicago UFS model used MIRs in conjunction with abundance to estimate vector index. For the California Risk Assessment model, mosquito abundance is compared to the 5-year average for the same area and time period. Viral infection rates are expressed as either MIRs [51] or MLEs [52] per 1,000 female mosquitoes tested. Due to differences in the attractiveness of traps to different subsets of the population, abundance and infection prevalence data are not pooled across trap types, but the most sensitive trap type’s value is used in the risk assessment. Also, due to differences in the sensitivity of traps between species and spatial heterogeneity in the distribution of *Culex tarsalis* and *Cx*. *pipiens* complex mosquitoes relative to humans, separate risk calculations for each species are suggested. The Temperature-trait-based Relative R_0_ model does not incorporate surveillance data as currently structured.

The 2 Model-EAKF Systems include human case data from the current year, unlike virtually all the other models (the California Risk Assessment allows it to be incorporated, but this is generally not done in practice). Historical human case data are used by several of the models, including the RF1, ArboMAP, the Chicago UFS, and the NE_WNV County-years models. In addition to training on past estimates of human cases, the NE_WNV County-years model included the rate of cumulative incidence of human cases as the total number of previous cases, for each county and each year, per 100,000 population on the basis that previous exposure to WNV reduces human infection rates [53]. The Chicago UFS model included human cases following a zero-inflated Poisson distribution.

#### Climate data inputs

While many models used climate data, models were constructed with different climate data sources. The Spatial Risk Random Forest model was based on 4 km gridded data from the Precipitation elevation Regressions on Independent Slopes Model (PRISM) [54,55]. Time series of weather data used in ArboMAP are typically obtained from gridMET [56] through Google Earth Engine (GEE) [57] using a custom downloader script [58]. gridMET combines data from both PRISM [55] and NLDAS-2 [59] into a single high-resolution gridded data set. The GLMER Ensemble model used approximately 13 km^2^ gridded monthly averages in temperature, precipitation, specific humidity, and soil moisture [59] from the North American Land Data Assimilation System (NLDAS) Mosaic submodel data set. The RF1 model used soil moisture data from the NLDAS Noah submodel data set, and initially with a published ensemble of temperature and precipitation data [60], and later with data from gridMET as above. The NE_WNV County-years model used lags of drought (1-month Standardized Precipitation Index (SPEI); 1-month Standardized Precipitation and Evapotranspiration Index) [61] and temperature variables (standardized temperature deviations from the mean, standardized precipitation deviations from the mean, from NOAA’s Climate Divisional Database) [62]. The Harris County model used the mean, standard deviation, and kurtosis for temperature and rainfall from local weather stations. While the Temperature-trait-based Relative R_0_ Model requires a temperature input, the model is flexible with respect to the choice of temperature input. The Temperature-forced Model-EAKF System used a mean climatology based on NLDAS-2 data from 1981 to 2000 and each outbreak year for each region of interest.

#### Model selection approaches

The GLMER Ensemble approach, the Harris County Model, and the NE_WNV County-years model all used the Akaike information criterion (AIC) for model selection. In the Harris County and NE_WNV County-years models, the model that minimized the AIC score was selected. In the GLMER Ensemble, all combinations of predictor variables were considered. Those models for which all explanatory variables were significant with 95% confidence were ranked by AIC [63,64]. The Akaike weight was calculated, and the set of models whose Akaike weights sum to 0.95 were used for the inference. The RF1 model used a two-stage fitting process for the Random Forest, removing all variables below a calculated mean importance score, and then removing additional variables that did not increase the model’s explanatory power using a variance partitioning approach [28]. The Spatial Risk Random Forest model did not use variable selection and included all predictor variables in the inference. The Model-EAKF System optimizes a 300-member ensemble of model simulations and, in so doing, provide an improved, posterior estimate of the true state as well as estimates of unobserved state variables and parameters. Model-EAKF System forecasts were repeated 10 times with different randomly selected initial conditions and evaluated for accuracy according to prescribed forecast metrics.

## Discussion

We qualitatively compared the models in the context of 6 potential decisions related to public health and vector control response to WNV (Table 5). This qualitative comparison was made based on the scale of the model and the scale of the decision. We found that some models, such as those developed at the coarser spatial and temporal scales (i.e., county/annual), are not useful for many of the decisions needed for vector control operations. Indeed, only 3 out of 13 models (the Temperature-trait-based Relative R_0_ model, the California Risk Assessment, and the Chicago UFS model), would be potentially capable to guide spatial and temporal adulticiding based on model resolution. However, the Temperature-trait-based Relative R_0_ model had not been developed or validated for this purpose. The California Risk Assessment model provides a threshold-based risk but does not quantitatively predict the number of human cases in the present year.

The models reviewed here included 3 different classes of models. Nearly all of the models have been implemented in the statistical software R [65]. Most of the models reviewed used human data as an input with 4 exceptions (the Temperature-trait-based Relative R_0_ model, the GLMER Ensemble, the Harris County model, and the California Risk Assessment as applied for real-time decisions by most vector control agencies). Climate was also a common input for models, having been used in all models except the Model-EAKF System (but note there is a temperature-forced version) and the null models. This is unsurprising, given the importance of climatic conditions on the mosquito life cycle (e.g., [66]). Landcover, sociological inputs, and other inputs were less common, even though these inputs may also be important in understanding disease dynamics (e.g., [67,68]). Model outputs were more heterogeneous, making comparisons across models more challenging. Most models focused on either annual cases or seasonal mosquito infection rates, or both. Fewer models examined patterns within a season, notably the ArboMAP model, the Chicago UFS model, the Harris County model, the 2 Model-EAKF System models, and the California Risk Assessment. Model applications are difficult to compare qualitatively and will be best examined through quantitative comparisons on common data sets, as R^2^ values and RMSE values can be difficult to interpret across scales and in the context of different numbers of cases or infection rates. Most of the models have been applied with nonoverlapping domains, making direct comparisons more difficult (Fig 1). Some of the models are specific examples of more general approaches that can be applied at finer scales.

Many of the models were designed to give an indication of whether it will be a “good year” or a “bad year” for WNV, without a direct, specific connection to decisions related to WNV control (e.g., Models E, F, G, I; Table 1). Temperature and precipitation may play a large role in such determinations [69], and such relationships may be the complex result of several interacting traits [27]. None of the models directly address the question of initial vector or viral surveillance, although such surveillance could be guided by spatial risk, in which case models A to H could be used to help guide general regions. The California Risk Assessment (M) provides recommendations for enhanced surveillance as risk levels increase. The Model EAKF Systems (I and J) specify specific quantitative surveillance requirements in order to be implemented but do not address the question of where such surveillance should take place.

Broadly, many of these models were developed within a local context. As a consequence, they are not necessarily the “best” model, but one sufficient to the task. In addition, being highly local means the models may be difficult to generalize to new locations. Regional variation is expected in the underlying processes. In some cases, the models are very closely tied to a specific region (e.g., the Chicago UFS model and the GLMER Ensemble) and influenced by variability in surveillance programs (spatiotemporal resolution). Regions vary in the dominant mosquito vector(s), the degree to which they are rural or urban, human risk-taking behavior (e.g., time spent outdoors, presence of window screens, and presence of mosquito breeding habitat), mosquito surveillance, and human socioeconomic status and ability to report biting mosquitoes.

Our analyses demonstrated that there is no “one size fits all” model—different models may be needed to guide the vector control and public health decisions considered here. Some decisions are made early in the season, while others are made later in the season. Decisions during the season may be constrained by planning made prior to the mosquito season. The decisions also vary on the spatial scale at which they take place, with public outreach taking place potentially across an entire state, while truck-based or aerial insecticide applications take place on localized scales of up to several square kilometers. It is important to note that these decisions are informed by WNV risk but are also influenced by social factors (e.g., interest in vector-borne disease around outdoor activities [70] or specific holidays such as Memorial Day or Fourth of July), financial (e.g., budget constraints) [70], environmental (e.g., current weather), and regulatory/political factors (e.g., protected ecosystems and restrictions on adulticide applications and willingness to pay for control) [70–73]. Vector control operations in the US are highly localized, and substantial regional variation exists in the timing of decisions, the thresholds used for decisions, and the willingness to apply adulticides. For example, Harris County, TX is primarily managed by a single agency (Harris County Public Health Mosquito and Vector Control Division, although several small cities and municipalities will also control nuisance mosquitoes) covering a geographic area of approximately 4,600 km^2^. Cook and DuPage Counties, Illinois have 4 mosquito abatement districts covering a geographic area of almost 3,500 km^2^.

WNV case incidence rates also vary from region to region. A change in the associated causal factors would likely influence the particular model’s performance. Regions also differ in their surveillance efforts, surveillance methods, and trap density. These factors affect the quality of the data going into the models and the quality of the data being used to evaluate the model. For example, a model could do a very good job of predicting the “true value,” but with poor data, the model may be scored worse than a model that predicted an observed prevalence that was consistent with biases created through data collection or sampling. Regions also vary in their turnaround times for data [39], and this may influence the degree to which different models can be implemented. Thus, more than one model may be necessary, and models may need to accommodate additional location-specific constraints.

In addition, the workshop discussions highlighted the importance of quantifying the value of information associated with model results. In many mosquito abatement districts, larvicide and public outreach are routine actions and are unlikely to be strongly affected by variations in the predicted risk of WNV. Other districts may dynamically increase public health outreach or larvicide application during “bad” years. In contrast, decisions regarding adulticide application are usually made based on perceived risk at a given point in time. However, these decisions typically take place on scales below the spatial resolution of most models. In practice, adulticide applications may be reactive to positive detection of WNV in mosquitoes, birds, or humans or a specified metric such as MIR or the vector index. While some of the models used vector index as an input, none of them included vector index as a predicted output (Table 3), despite common use of this metric by vector control. Increased temporal and spatial resolution in model results and a focus on these quantities could make models more applicable to these decisions. As it stands, most models are parameterized on the county scale or larger, and this prevents them from being utilized for decisions at local scales. In part, this is driven by the availability of data—human case data in particular are difficult to obtain at scales finer than the county because of privacy concerns.

Models may also be limited by accuracy thresholds needed for decisions. Often, while models may provide more information than a null model, these models may not provide enough confidence to be used for decision-making. On the other hand, relying on null models for early warning of upcoming high-risk events is not feasible either. Trade-offs between confidence in imperfect model predictions of extreme events and uninformative priors (e.g., null models) have to be made. In case of rare events, the uncertainty is usually higher than for more regular events. Some of this may be related to inadequate data—for rare events, especially large data sets may be needed for model training. Models may also be limited by heterogeneity in underlying processes. Models are typically aggregated over multiple trap sites, with the assumption that similar processes are operating at all trap sites. If traps differ strongly from one another in the underlying mosquito or disease dynamics, this heterogeneity may be averaged over during the modeling process and lead the model to produce mean predictions that are incorrect for all locations. Improved models for identifying regions of homogenous risk could aid in this aggregation process.

Incorporating the effects of public health interventions such as vector control efforts into models may be difficult as well. Interventions, even when applied at discrete locations, typically have effects that extend beyond the place and time of treatment that are not easily quantified. Also, most interventions do not have suitable controls, as interventions are required to protect public health. Therefore, finding a control site with no intervention that is equivalent to the treatment area is difficult (as any sites with equal risk would likely be treated). Before–after controls are challenging, as mosquito populations can be dynamic. For example, even if populations do not decrease after an intervention, it is unclear whether the intervention did not work, or whether the mosquito populations would be much higher in the absence of the intervention.

### Future directions

To improve the quality of modeling for decision-making, a clear mapping between model outputs and information needed for decisions would be beneficial. Quantifying the gain of information achieved by the model and the value of that information gain would provide clear guidance for when to apply a model to guide decision-making. Metrics, such as human cases averted, or resources saved due to an early intervention, could strengthen the justification for decisions made on the basis of a model. These metrics would need to be carefully described, however, as it may be difficult to know exact numbers of cases averted, and, therefore, language should reflect uncertainty in the results.

Formal quantitative comparisons of existing models may be useful to ensure that all decision-makers are able to select the best models for their regions and the decisions they need to make. Multiple models exist at the county-annual scale (Figs 4 and 5), and comparisons could be performed for 4 of the 6 decisions (Table 5). Formal model comparisons have been performed for dengue [74] and leishmaniasis [75]. A comparison of linear and classification and regression tree analysis methods has been performed locally for WNV [76]. Models for spatial risk, models for “good” versus “bad” years, and models that guide local decisions such as application of adulticides could all be compared separately. Quantitative comparisons would provide a degree of rigor and could also contribute to assessing the gain of information associated with each model. A formal quantitative comparison should consider the lead times associated with each model in the context of the lead times needed for control efforts [77]. In addition, the creation of standard data sets for each key output would aid in model comparison. Standard data sets are used in machine learning (e.g., the Anderson’s Iris data) [78] and provide a basis for comparing different methods. This will be a challenging task given the complexity and regional variation of the disease system.

There is a critical need for more social science research, particularly the need to incorporate human behavior related to vector control and exposure risk in the models. Mosquito transmission takes place within social-ecological systems [79]. Integrated mosquito management (IMM) [80] in part aims to influence human behavior and the interaction of humans and mosquitoes. Predictive computational models of human behaviors [81] are potentially powerful tools to support IMM interventions (e.g., source reduction, public education, and community involvement). This is particularly true if the models can link the individual attributes and behaviors with the dynamics of the socioenvironmental systems within which individuals/residents operate [81]. Such considerations as well as making available actionable, customizable (e.g., predictive analysis), and easy-to-use model outputs can also encourage mosquito control practitioners to use such model outputs for local level decision-making [71]. In addition, models should be culturally responsive to the needs of state, tribal, local, or territorial public health and mosquito control agencies.

Finally, models need to be based on sound scientific data. A recent study identified over 1,000 mosquito control agencies in the continental US. Of these, 152 agencies had publicly available open access mosquito data sets, while 148 agencies had live data that can be leveraged and used with good effect [82]. Indeed, improved integration of IMM interventions such as public health campaigns, larvicide applications [83,84], and adulticide applications [85,86] into the models will be critical to assessing the role of interventions in a modeling framework.

## Supporting information

S1 Text Model description template. (DOCX)

S2 Text Model description instructions. (DOCX)

S3 Text Regional decision-makers template. (DOCX)

S4 Text Detailed model descriptions. (DOCX)
