# Supplementary material for: A proposed framework for the development and qualitative evaluation of West Nile virus models and their application to local public health decision-making
Source: PLoS Negl Trop Dis. 2021 Sep 9;15(9):e0009653. doi: 10.1371/journal.pntd.0009653 (PMC8428767; doi:10.1371/journal.pntd.0009653)
Supplement: S1 Text — (DOCX) [file pntd.0009653.s001.docx]

S1_text from: “A proposed framework for the development and qualitative evaluation of West Nile virus models and their application to local public health decision-making”

**Model Description Template**

**(see S2_text for an explanation of the Model Description and Table fields)**

**Model Description**

**<Paragraph describing your modeling approach here>**

**<key details to include: Type of underlying model (e.g., linear model, Random Forest)**

**<additional processing applied (e.g., converting positive pools to maximum likelihood estimates of mosquito infection rates>**

**<model selection approach, if applicable>**

**<Any specific data requirements, such as minimum sample sizes, required distributions of underlying data, or level of quality needed to achieve accurate results>**

**<Any additional prediction targets not covered by Table 3>**

**<Computational resources required to run model>**

**<Anything else you would like researchers/users to know about your model>**

**Table 1: Model Overview**

| Model | Class of Model | Spatial Resolution | Temporal Resolution | Software | Code Available |
| --- | --- | --- | --- | --- | --- |
|  |  |  |  |  |  |
|  |  |  |  |  |  |

**Table 2: Model Inputs**

| Model | Human Data | Mosquito Surveillance | Other Surveillance | Climate / Weather | Land-cover | Socio-logical | Other |
| --- | --- | --- | --- | --- | --- | --- | --- |
|  |  |  |  |  |  |  |  |
|  |  |  |  |  |  |  |  |

**Table 3: Model Output / Predictions**

| Model | Annual Cases | Seasonal MLE/ MIR | Peak MLE/ MIR | Peak Week for MLE/ MIR | Peak Week (cases) | Probabil-istic? | Additional Prediction Targets |
| --- | --- | --- | --- | --- | --- | --- | --- |
|  |  |  |  |  |  |  |  |
|  |  |  |  |  |  |  |  |

**Table 4: Model Applications**

| Model | Study | Prediction Target | Sample Size | Spatial Domain | Time Domain | Testing  Method | Metric | Metric Score |
| --- | --- | --- | --- | --- | --- | --- | --- | --- |
|  |  |  |  |  |  |  |  |  |
|  |  |  |  |  |  |  |  |  |

**Literature Cited**
