# Supplementary material for: A proposed framework for the development and qualitative evaluation of West Nile virus models and their application to local public health decision-making
Source: PLoS Negl Trop Dis. 2021 Sep 9;15(9):e0009653. doi: 10.1371/journal.pntd.0009653 (PMC8428767; doi:10.1371/journal.pntd.0009653)
Supplement: S2 Text — (DOCX) [file pntd.0009653.s002.docx]

S2_text from: “A proposed framework for the development and qualitative evaluation of West Nile virus models and their application to local public health decision-making”

**Model Description Information**

**Model Description**

The model description is a written description of the model. This should be a brief explanation of the model, including the core elements of the model (statistical approach, such as a general linear model, mathematical approach, etc.), any processing applied to the standard data inputs (see Data Sharing Agreement) in order to run the model, such as converting positive mosquito pools into an estimate of mosquito infection rate, and any model selection applied as part of the modeling process. In addition, any key data requirements should be listed – for example, if the underlying data needs a specific distribution, or if there is a minimum sampling effort required to parameterize the model. Any additional prediction targets not listed in Table 3 should also be described here. A brief description of the computational resources required to run the model should be mentioned, as well as any encountered limits. Please also include any additional details that you think are relevant and are not addressed by the above

**Model Overview Fields**

- Model Class
  - Choose one of the following:
    - Spatial Patterns
      - Models with predictions that do not vary by year
    - Early Warning
      - Models that do not include current-year surveillance data
      - May include current year climate/weather data
      - Model lead time on the order of days to months
    - Early Detection
      - Models that include current-year surveillance data
      - May include other data streams
      - Model lead time on the order of days to months
  - This field follows Barker’s [1] classification of models.
- Spatial Resolution
  - At what resolution does the model run?
    - E.g., Point, 12 x 12 Grid, County Subdivision, County
- Temporal Resolution
  - At what time step does your model run?
    - E.g., static, annually, monthly, biweekly, weekly, daily
      - Static would be used for models of spatial pattern, that do not update over time
- Software
  - The software used for running the model (e.g., R) including any specific packages required
- Code Available
  - If code is available, add a link to code, or N if code is not publicly available.

**Model Inputs Fields**

- Human Data
  - Does the model use historical human data as an input?
    - Y for YES
    - N for NO
  - NOTE: For the purposes of this table, this does not include current-year human case reports, those should be included in “Other surveillance
    - Historical data are expected to be relatively complete and uniform, while real-time data are expected to be in-progress, and have heterogenous availability.
- Mosquito Surveillance
  - Does the model use mosquito surveillance data as an input
    - Y for YES
    - N for NO
  - If a subset of mosquito surveillance is used, please describe in the model description paragraph – e.g., if the model restricts to only gravid trap data, or only specific mosquito species.
- Other Surveillance
  - Does the model use other surveillance data, such as current-year human case data, sentinel chicken data, dead bird counts?
    - Y for YES
    - N for NO
  - If YES, please list and describe in the Model Description paragraph
- Climate / Weather
  - Does the model use Climate / Weather data?
    - Y for YES
    - N for NO
  - If YES, please describe the data requirements in the Model Description Paragraph.
  - Please include dynamic hydrological data, such as water table levels and soil moisture here. Hydrological variables related to physical features, such as distance to nearest water or stream, should be included in the Landcover category.
- Landcover
  - Does the model use Landcover data?
    - Y for YES
    - N for NO
  - If YES, please describe the data requirements in the Model Description Paragraph.
- Sociological
  - Does the model use Sociological data?
    - Y for YES
    - N for NO
  - If YES, please describe the data requirements in the Model Description Paragraph.
  - Examples include human population, population density, age, ethnicity, income information, percent of population with air conditioners, presence of a septic system, etc.
- Other
  - Does the model use a data stream not described above?
    - Y for YES
    - N for NO
  - If YES, please describe the data requirements in the Model Description Paragraph.
  - Examples include avian host densities, numbers of wastewater treatment plants

**Model Outputs / Predictions Fields (Does the model produce the following outputs?)**

- Annual Cases
  - Total Human Cases by the spatial resolution unit for the year.
  - Y for YES; N for NO
- Seasonal MLE / MIR
  - An estimate of the Seasonal MLE / MIR.
  - Y for YES; N for NO
  - The date range bounding the model’s seasonal estimate needs to be reported, and the model’s ability to correct for varying sampling effort over the season should be described in the Model Description Paragraph.
- Peak MLE / MIR
  - An estimate of the peak mosquito infection rate
  - Y for YES; N for NO
  - If YES, please define the date range or other criteria to delimit the peak in the Model Description Paragraph
- Peak Week for MLE / MIR
  - An estimate of the timing (week) of the peak MLE / MIR
  - Y for YES; N for NO
- Peak Week (cases)
  - An estimate of the timing (week) of the peak number of human cases
  - Y for YES, N for NO
- Probabilistic
  - Y for YES
  - N for NO
- Additional Prediction Targets
  - Y for YES
  - N for NO
  - If YES, please describe in the Model Description paragraph

(Continues with Model Application Fields on next page)

**Model Applications Fields**

- Study
  - Author and year of a publication describing the application (if applicable). Full citation should appear in Literature Cited section.
- Prediction Target
  - What Model Output was predicted (see Table 3)?
  - Please use a separate line for each Prediction Target.
- Sample Size
  - How many spatial-temporal units were used in the analysis?
- Spatial Domain
  - Over what spatial extent was the model applied?
- Time Domain
  - Over what time period was the model trained?
- Testing Method
  - The method used to evaluate the model
  - E.g., Fit to training data only, Data split into training and testing data and fit reported from testing data, fit to novel testing data not used in model development, Leave-one-out cross-validation, Leave-one-year-out cross-validation
  - Please describe the testing method in the Model Description Paragraph
- Metric
  - R^2^
  - RMSE
  - CRPS
  - Other
    - If another criterion is used, please describe in the Model Description Paragraph
  - None
    - Only use if model was not evaluated as part of the application
- Metric Score
  - Please give the score associated with the selected Metric criterion

**Resources for Standardized Reporting**

- STROBE: <https://www.equator-network.org/> [2]
- Model Evaluation: [3] <https://www.sciencedirect.com/science/article/abs/pii/S0304380013005450?via%3Dihub>
- Model Reporting: [4] <https://www.sciencedirect.com/science/article/abs/pii/S0304380014000611>
- Overview, Design concepts, and Details Protocol: [5]
- R Open Sci Package guidelines: <https://devguide.ropensci.org/>

(References on next page)

**References**

1. Barker CM. Models and Surveillance Systems to Detect and Predict West Nile Virus Outbreaks. J Med Entomol. 2019 [cited 27 Sep 2019]. doi:10.1093/jme/tjz150

2. Von Elm E, Altman DG, Egger M, Pocock SJ, Gøtzsche PC, Vandenbroucke JP. The Strengthening the Reporting of Observational Studies in Epidemiology (STROBE) statement: guidelines for reporting observational studies. Annals of internal medicine. 2007;147: 573–577.

3. Augusiak J, Van den Brink PJ, Grimm V. Merging validation and evaluation of ecological models to “evaludation”: a review of terminology and a practical approach. Ecological Modelling. 2014;280: 117–128.

4. Grimm V, Augusiak J, Focks A, Frank BM, Gabsi F, Johnston ASA, et al. Towards better modelling and decision support: Documenting model development, testing, and analysis using TRACE. Ecological Modelling. 2014;280: 129–139. doi:10.1016/j.ecolmodel.2014.01.018

5. Grimm V, Berger U, DeAngelis DL, Polhill JG, Giske J, Railsback SF. The ODD protocol: a review and first update. Ecological modelling. 2010;221: 2760–2768.
