# Supplementary material for: A proposed framework for the development and qualitative evaluation of West Nile virus models and their application to local public health decision-making
Source: PLoS Negl Trop Dis. 2021 Sep 9;15(9):e0009653. doi: 10.1371/journal.pntd.0009653 (PMC8428767; doi:10.1371/journal.pntd.0009653)
Supplement: S3 Text — (DOCX) [file pntd.0009653.s003.docx]

S3_text from: “A proposed framework for the development and qualitative evaluation of West Nile virus models and their application to local public health decision-making”

**Regional Decisions Template**

**Regional Description: <SPECIFY REGION HERE>**

< Please provide a brief description of the decisions made relating to West Nile virus in the region. Please describe the spatial location of the region in which vector-control activities take place, and any region-specific details important to vector control. A ‘region’ may be as large or small as one chooses, but ideally it should serve as a useful grouping for the decisions made within it (e.g., within a state, within a county, within a mosquito abatement district, within several mosquito abatement districts with similar policies). Please also define any additional forecasting outputs used that are not predefined in Table 2.>

**Table 1: Stakeholder Overview.**

| Stakeholder^1^ | Type of Stakeholder^2^ | Scale of Operation^3^ |
| --- | --- | --- |
|  |  |  |
|  |  |  |
|  |  |  |
|  |  |  |
|  |  |  |

^1^Stakeholder refers to organization or agency making the decision. Example stakeholders include Mosquito abatement districts, county health departments, City officials, state health departments, state legislatures, state executives, the general public.

^2^ A classification for the stakeholder: Federal Government, State Government, Local Government, Local Vector Control, General Public

^3^ The scale at which the stakeholder operates: State, County, County Subdivision, Census Tract, Household

**Table 2: Decisions Overview**

| Stakeholder^1^ | Decision^2^ | Spatial Extent of Decision^3^ | Timing of Decision^4^ | Timing of Application^5^ | Constraints affecting Decision^6^ |
| --- | --- | --- | --- | --- | --- |
|  |  |  |  |  |  |
|  |  |  |  |  |  |
|  |  |  |  |  |  |
|  |  |  |  |  |  |
|  |  |  |  |  |  |
|  |  |  |  |  |  |
|  |  |  |  |  |  |

^1^ Same as Table 1

^2^ A Decision made by the stakeholder related to West Nile virus. These may include mosquito surveillance, public health campaigns, mosquito larvicide control, truck-based adulticide application, aerial adulticide application, use of personal protective behaviors by general public, vector control budgets, restrictions on households providing mosquito breeding habitat, restrictions on activities

^3^ The spatial extent affected by the decision

^4^ The time when the decision is typically made. Please be as precise as is practical. This can be in terms of absolute time (e.g., CDC week 24) or relative time (e.g. 2 weeks after first WNV-positive pool)

^5^ The time when the decision is typically applied. Please be as precise as is practical. This can be in terms of absolute time (e.g., CDC week 24) or relative time (e.g. 2 weeks after first WNV-positive pool)

^6^ Please indicate categories of constraints that may limit a decision here, and please describe these constraints in the Regional Description above. Categories are: Budget, Weather, Resource, Political, Other. *Budget*: limited due to insufficient funds to implement it. *Weather*: limited due to adverse weather conditions. *Resource*: limited due to unavailability of resources (e.g., there is only one company that does aerial sprays and they have no availability). *Political*: limited due to laws or other legal restrictions (e.g., legal prohibitions against adulticide use). *Other*: a constraint not addressed by the preceding.

**Table 3: Quantitative information regarding decision-making**

| Stakeholder^1^ | Decision^1^ | Default Action^2^ | Information needed for decision^3^ | Decision based on a threshold or metric?^4^ | Accuracy required for Decision^5^ |
| --- | --- | --- | --- | --- | --- |
|  |  |  |  |  |  |
|  |  |  |  |  |  |
|  |  |  |  |  |  |
|  |  |  |  |  |  |
|  |  |  |  |  |  |
|  |  |  |  |  |  |
|  |  |  |  |  |  |

^1^ Same as in Table 2

^2^ The default or ‘normal year’ action taken

^3^ Please select from one of the following forecast outputs. If the information needed is not captured by a forecast output, enter a new forecast output with a definition given in the regional description above. Forecast outputs include: annual human cases, seasonal mosquito infection rate, peak mosquito infection rate, timing of peak mosquito infection rate, weekly human cases, weekly mosquito infection rate, vector index (mosquito abundance times mosquito infection rate), mosquito abundance

^4^ Is there any specific threshold or numerical value that causes a decision to be made? If it is concise, please enter it here, or enter NO if there is no value. Otherwise, enter YES here and please describe the criteria used to make the decision in the Regional Description.

^5^ How accurate does information need to be in order to change the default decision?
