# Supplementary material for: A proposed framework for the development and qualitative evaluation of West Nile virus models and their application to local public health decision-making
Source: PLoS Negl Trop Dis. 2021 Sep 9;15(9):e0009653. doi: 10.1371/journal.pntd.0009653 (PMC8428767; doi:10.1371/journal.pntd.0009653)
Supplement: S4 Text — (DOCX) [file pntd.0009653.s004.docx]

S4_text from: “A proposed framework for the development and qualitative evaluation of West Nile virus models and their application to local public health decision-making”

**Model descriptions**

**Spatial Risk Models**

*A. Historical Null Model*

The Historical Null Model is a simple probabilistic null model based on historical occurrences of annual human cases or seasonal mosquito infection rates; this model could be adapted to any forecast target. The historical null model samples with replacement from historical observations to give probabilistic predictions of the outcome in the current year. A benefit of the model is that it makes no assumptions about the distribution of WNV cases other than assuming that the past will be predictive of the future. This gives the null model flexibility to represent bimodal or multimodal distributions, but a weakness is that it may also generate unrealistically discontinuous predictions (e.g., if there historically have been 1, 2, 3, and 5 human cases in a location, the model will assign a probability of 0% to 0, 4, or 6 human cases, and ~25% to 1, 2, 3, and 5 cases). As a consequence, the model will not respond in a predictive manner to novel or changing situations, nor average over discontinuities in the data. As the model predictions are based on historical observations, the model is expected to perform better with an increased number of historical observations to draw upon. The model is unlikely to be very useful at fine or very-fine spatial resolution or daily temporal resolution, where it effectively will always predict no cases of West Nile virus. The model is computationally efficient, and runs quickly (within minutes) on a standard desktop PC.

*B. Spatial Risk Random Forest Model*

The Spatial Risk Random Forest Model [1] aimed to identify the seasonal climate variables that influence the spatial extent and magnitude of WNV incidence in humans. The authors created a random forest model [2] of present day mean annual WNV incidence using county-level CDC case reports from 2005-2018 and monthly-mean surface air temperature and precipitation at 4 km resolution from Precipitation elevation Regressions on Independent Slopes Model (PRISM) [3,4]. Total human case counts of WNV, including both neuroinvasive and non-neuroinvasive disease, were obtained at the county-level by year for the conterminous US from 2005-2018 from the US Centers for Disease Control and Prevention. Case counts were converted to WNV incidence (cases per 100,000 population) using annual county-level population estimates [5,6]. To compare climate with the county-level WNV incidence data, the gridded climate data were spatially averaged to the county-level using county shapefiles from the US Census Bureau (https://www.census.gov/geo/maps-data/data/tiger-line.html). Three-month mean seasonal climate variables were calculated from monthly observations during 2005-2018 for direct comparison to the WNV dataset. The different seasons used for averaging the climate information were winter (DJF), spring (MAM), summer (JJA), and autumn (SON).

The mean Spatial Risk Random Forest Model was created by running the random forest model [7] in R [8] 500 times, each time on a different, randomly selected set of training data, with each iteration of the model generating 1,000 separate regression trees. Three randomly selected predictor variables were candidates at each split in the Spatial Risk Random Forest Model. To avoid overfitting, a minimum node size of 5 was used, so that each terminal node (i.e., leaves) in the regression trees described at least 5 counties. The estimated variable importance and node purities were averaged across the 500 model iterations. The importance of each climate variable was measured in the model by calculating the decrease in modeled mean square error from randomly permuting each predictor variable. Model selection was not used to reduce the number of climate input variables since the number of variables was much smaller than the number of counties modeled and there was no scientific basis for not considering a variable-season combination. The out-of-sample county-level estimations of WNV incidence from the 500 Spatial Risk Random Forest Models were averaged to create the mean Spatial Risk Random Forest Model and for reporting the final estimation of county-level mean annual WNV incidence.

The R^2^ performance of the Spatial Risk Random Forest Model averaged across the 500 iterations is 0.67 [0.64-0.70] for the in-sample training data and 0.59 [0.44-0.70] for the out-of-sample testing data. The RMSE performance of the Spatial Risk Random Forest Model averaged across the 500 iterations is 3.3 cases per 100,000 population per year for the in-sample training data and 3.7 cases per 100,000 population per year for the out-of-sample testing data (compared to the county-level average incidence at 2.3 cases per 100,000 population per year [median 0.3, range 0.0-50.7]). The model can be run on a personal machine in approximately 5 minutes.

*C. Temperature-trait-based Relative R_0_ Model*

The Temperature-trait-based Relative R_0_ Model is a set of three temperature-dependent, trait-based transmission models for WNV transmitted by *Culex pipiens*, *Cx. quinquefasciatus*, and *Cx. tarsalis* [9]. Here relative R_0_ is an index of relative risk of WNV transmission. The model was built by fitting nonlinear thermal response curves to data on mosquito and virus traits from lab experiments in constant temperature environments. These trait responses were then used in a modified Ross-McDonald equation for transmission. The model can be used to make spatial or temporal predictions at any resolution. Typically, these types of models are used to make spatial predictions, including for various climate change scenarios and future timepoints [e.g., as was done in 10 for the malaria parasite *Plasmodium falciparum*]. The only model input is temperature (°C). The model output is relative *R_0_* (i.e., *R_0_* scaled between 0 and 1 to the maximum level of transmission allowed by temperature). The model was validated using a Generalized Additive Model fit to county-level human case data and summer temperatures averaged over 16 years (to minimize the impact of immunity and precipitation on interannual variation). The GAM found a clear unimodal response with maximum incidence at average summer temperatures of 24°C, a close match to the trait-based models that predicted maximum transmission at 24-25°C.

**Fig. S4.C.1.** Trait-based transmission models and observed human cases of West Nile disease both show a unimodal response to temperature with transmission maximized at 24-25°C. A) Transmission risk (as relative R_0_) predicted by the Temperature-trait-based Relative R_0_ Model parameterized for *Culex pipiens* (solid line), *Cx. quinquefasciatus* (dashed line), and *Cx. tarsalis* (dotted line). B) Generalized Additive Model (GAM, grey line) with 95% CIs (grey shading) fit to county-level human case data and summer temperatures averaged over 16 years. County-level means (black points) are binned (42 counties per bin; error bars are SE) for visual clarity. GAM predictions are restricted to the range of observed average summer temperature values (7.1–31.6°C). Modified from [Shocket et al. 2020] under CC BY 4.0 (https://creativecommons.org/licenses/by/4.0/).

*D. Spatial Risk High Resolution BRT Model*

The spatial risk high-resolution Boosted Regression Tree (BRT) model was developed to identify environmental covariates of human WNV cases in South Dakota and generate a high resolution (300 m grid cells) map of disease risk for the entire state [11]. Human case data were obtained from the South Dakota Department of Health and included geocoded home addresses of 1,378 WNV cases reported in 2004-2017. We used the technique of Chuang et al. [12] to generate control points that represented the background distribution of the human population. Data from all years were combined to model the overall geographic distribution because previous WNV research found that geographic clusters have remained relatively stable over time [13]. We examined three groups of environmental predictors: 1) remotely-sensed spectral indices from the Moderate Resolution Imaging Spectroradiometer (MODIS) on board the Terra and Aqua satellites, 2) climate variables from the North American Land Data Assimilation System (NLDAS), and 3) land cover and physiographic from the National Land Cover Database (NLCD), National Wetlands Inventory (NWI), Soil Survey Geographic Database (SSURGO), and National Elevation Dataset (NED). Data were randomly assigned to training (80%) and validation (20%) sets for accuracy assessment, and this process was repeated 20 times. A combined model using all types of environmental variables had the highest mean AUC of 0.727. The models driven by climate (0.719), land cover and physiography (0.711), and spectral indices (0.679) had lower accuracy. Elevation, late-season humidity, and early-season satellite moisture indices were the most important environmental predictors. Climate and spectral indices that measured interannual variability were more important than those based on means or medians. The final model was used to generate a high-resolution map that predicted the ranked relative risk of WNV in South Dakota as a continuous variable between zero and one.

**Early Warning Models**

*E. RF1 Model*

The RF1 model [14] uses a Random Forest statistical model [2] in a two-staged approach. In the first stage, a model is fit with all relevant predictors, using 5,000 trees (default). Predictors can include human population and demographic data [15], landcover data [16], temperature, and hydrological data [e.g., 4,17], among others. The number of variables used at each split was determined empirically by trying each number of variables (from 1 to K, where K is the number of input variables) and using the parameter that provided the best fit. Node size used the randomForest package default of five [7]. In the second stage, the model was refit, with only predictor variables with mean importance scores above the mean importance score. The model was further refined through a variance partitioning approach [18] to identify a minimum predictive model. The model can be run for annual human cases, annual human incidence, or seasonal mosquito infection rate. For mosquitoes, the model uses maximum likelihood estimates of seasonal mosquito infection rate (MLE) calculated in R [19]. There are not specified minimum data requirements, but poor model results may result from insufficient training data. The model has been modified to produce probabilistic output using the quantReg R package [20,21]. The model can be run on a standard desktop PC for small regions (NY and CT), but will require additional refinement in order to run for the entire United States.


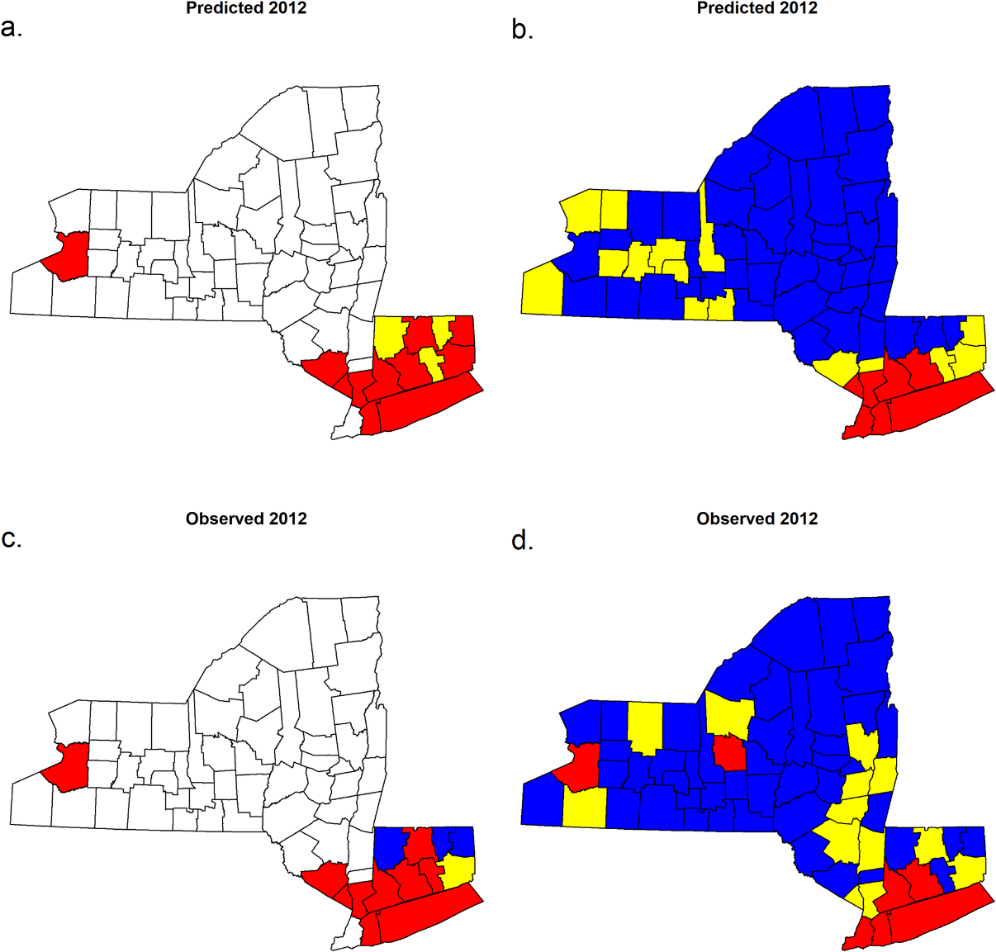


**Fig. S4.E.1.** Predicted and observed WNV mosquito infection rates (*MLE*, a, c) and human cases (b, d) for 2012, a particularly widespread WNV year. *MLE* thresholds from [22]: blue corresponds to *MLE* < 1 mosquito per 1000, yellow corresponds to *MLE* 1–5 per 1000, and red to *MLE* > 5 per 1000. White indicates excluded counties for which we did not have mosquito surveillance data. For human cases (b, d), blue indicates no human cases, yellow indicates 1–5 cases, and red indicates more than 5 cases. From [14], used under CC BY 4.0 (https://creativecommons.org/licenses/by/4.0/).

*F. NE WNV Model*

The Nebraska WNV model [23] uses a general additive model with thin-plate splines (the R package mgcv [24]) for non- parametric modeling of distributed lags of drought and temperature data, using restricted maximum likelihood estimation with a log link and negative binomial distribution. Natural-log-transformed population was used as an offset variable to directly model cases (neuroinvasive and non-neuroinvasive) per 100,000 people. Categorical fixed effects accounted for the unique effects of the county and year, using sum-to-zero contrast coefficients. The model uses lags of drought (1-month Standardized Precipitation Index, SPEI; 1-month Standardized Precipitation and Evapotranspiration Index, [25]) and temperature variables (standardized temperature deviations from the mean, standardized precipitation deviations from the mean; [26] using February as the start of the lagged data (i.e., the February value was lag 0, January was lag 1, December, lag 2, and so on). The model can explore lag lengths of 12, 18, 24, 30 and 36 months. The best model is selected using the Akaike Information Criterion [AIC, 27,28]. Counties with no cases in any years are excluded as outliers to ensure model convergence. The rate of cumulative incidence as the total number of previous cases, for each county and each year, per 100,000 population was included in the model on the basis that previous exposure to WNV reduces human infection rates [29]. See [23] for details. The models were found to be more accurate at predicting presence/absence than a prior-year null model, but generally worse than a prior-year null model for numbers of human cases [23].


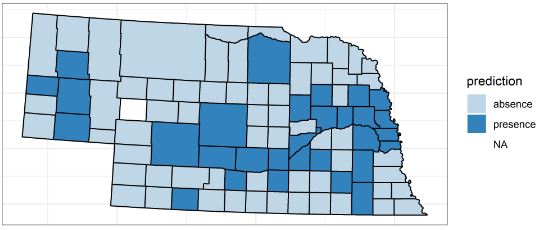


**Fig. S4.F.1** This map shows which Nebraska counties were predicted to have cases in 2018, based on models trained with data through 2017. “Presence” means they were predicted to have at least one case. Arthur County, white, is “NA” because it has never had any cases and was excluded as an outlier. From [23], used under CC BY 4.0 license (https://creativecommons.org/licenses/by/4.0/).


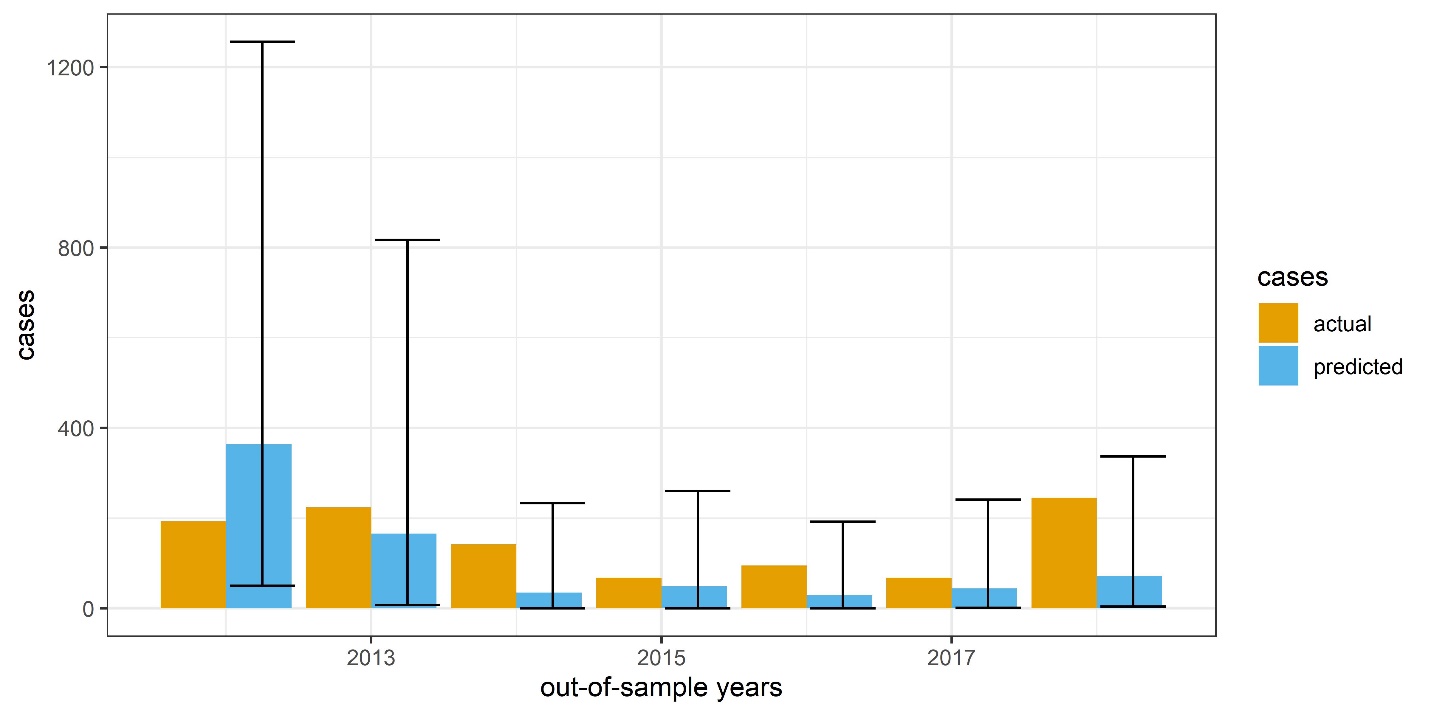


**Fig. S4.F.2.** Predicted vs. observed cases, with prediction intervals, for out‐of‐sample years, 2012–2018. This chart compares actual and predicted numbers of cases, with actual numbers in tan, and predicted numbers in blue, with prediction intervals. From [23], used under CC BY 4.0 license (https://creativecommons.org/licenses/by/4.0/).

*G. GLMER Ensemble*

WNV infection rates among mosquito vectors were modeled in Suffolk County, New York using readily available meteorological and hydrological conditions [22]. The North American Land Data Assimilation System (NLDAS) Mosaic submodel dataset was used to measure monthly averages in temperature, precipitation, specific humidity, and soil moisture [30]. These data are available at ~13 km^2^ spatial resolution. The *Culex* spp. WNV Infection Rate was calculated for each NLDAS Grid Cell and year. Meteorological and hydrological predictors were restricted to January-August to precede or coincide with WNV Infection Rates. All combinations of predictor variables were examined using a negative binomial mixed effects model with Grid Cell as a random effect to account for spatial variability. Multimodel inference was used to develop ensemble predictions. Models for which all explanatory variables were significant with 95% confidence were ranked by AIC [27,28]. The Akaike weight was calculated and the set of models whose Akaike weights sum to 0.95 were used for the inference. The model was validated using Leave-one-out temporal cross validation (LOOTCV) [a common modification of Leave-one-out-cross validation, 31]. Each year of data was iteratively omitted, and predictions were made using the data from the other years. Accuracy of model predictions compared with observed values was evaluated with Root Mean Square Error (RMSE).

**Early Detection Models**

*H. Harris County Model*

Mosquito abundance and WNV infection in mosquito pools from Harris County, TX were predicted with seasonally autoregressive forced models [32]. Seasonally autoregressive models are linear models that capture non-symmetric features in the seasonality of the underlying data. The mosquito infection rate was estimated at a monthly scale following the maximum likelihood method by Farrington [33]. Model development started by fitting a “Null” model with a seasonal autoregressive structure based on the inspection of the auto-correlation and partial auto-correlation function, which was used to pre-whiten covariates using a Kalman filter based on model coefficients [34]. In pre-whitening, any common seasonal or autoregressive structure in the focal time series is removed from the covariates, so that the observed correlation is not a product of a similar autocorrelation structure. Covariates included the mean, standard deviation, and kurtosis for temperature (8 stations) and rainfall (11 stations; 7 in common with temperature measurements) in the Harris county area and a time series of MODIS based NDVI and EVI [250 m resolution, 35]. Models were selected using AIC minimization [27,28] and validated using out of fit data using the predictive R^2^ [36].

*I. ArboMAP*

ArboMAP (Arbovirus Modeling and Prediction) is a software tool in R that allows a user to run and compare statistical models of human disease cases as functions of environmental and entomological indices. Models concern the district-week, which is either positive (=1) if there is at least one human case in that district (usually county) in that week or negative (=0) if there were no cases. ArboMAP uses logistic regression models with environmental indices (temperature, precipitation, humidity, etc.) included as distributed lags, with shapes governed by splines [37,38]. Time series of weather data are typically obtained from gridMET [39] through Google Earth Engine (GEE) [40]. Mosquito data are modeled before inclusion into the human model in their own mixed-effects models, in which exponential growth curves are imposed on mosquito infection rates in the early season. The estimated growth rate is then used as a covariate in the human models. These estimates of risk on a district-week basis are translated into estimates of human cases on district/state and week/year bases. Predictions of the burden of WNV cases over the entire transmission season are made every week beginning in June, and are summarized as risk maps and seasonal trends. The ArboMAP software was designed to facilitate WNV forecasting by epidemiologists working in state public health offices. The R script and an accompanying GEE web application automate most data acquisition and processing steps and generate formatted reports. ArboMAP has been used by the State of South Dakota since 2016 and is currently being implemented in Louisiana, Michigan, and Oklahoma.

*J. Chicago Ultra-Fine-scale Model:*

The Chicago Ultra-Fine scale (UFS) model is derived from a prior model [41]. The model is a logistic regression model with 1 km-wide hexagonal (spatial) and 1-week (temporal) resolutions. The model has a total of 82 covariates available for analysis at the “Ultra-fine-scale” (55 selected hexagons within the Northwest Mosquito Abatement District (NWMAD)), 59 covariates available for analysis at the “Local scale” (NWMAD, 1019 hexagons), and 40 covariates available for analysis at the “County scale” (Cook & DuPage Counties, 5345 hexagons). Covariates include environmental, land-use/land-cover, historical weather, light pollution, human socio-economic and demographic, mosquito abundance and infection, mosquito landing rates on humans, and human activity/exposure risk (the latter two covariates are available at the UFS only). In order to apply the model, all data need to be aggregated by week within the hexagonal grid (e.g., with ArcGIS, Redlands, CA; Environmental Systems Research Institute), and this can be a time-consuming and computationally-demanding process. Human cases entered into the model are assumed to follow a zero-inflated Poisson distribution, however, input covariates are expected to typically have non-zero values for each week (e.g., minimum infection rate and vector index, mosquito abundance, socio-economic status, etc.). The model may require considerable processing resources at the county scale, as >100 GB of data were used. Smaller subsets (e.g., the “local and ultra-fine-scale”) were substantially smaller in size (10 and 25 GB, respectively) and were processed on a PC with an i7 intel core processer (@3.6GHz) with 32 GB of RAM.


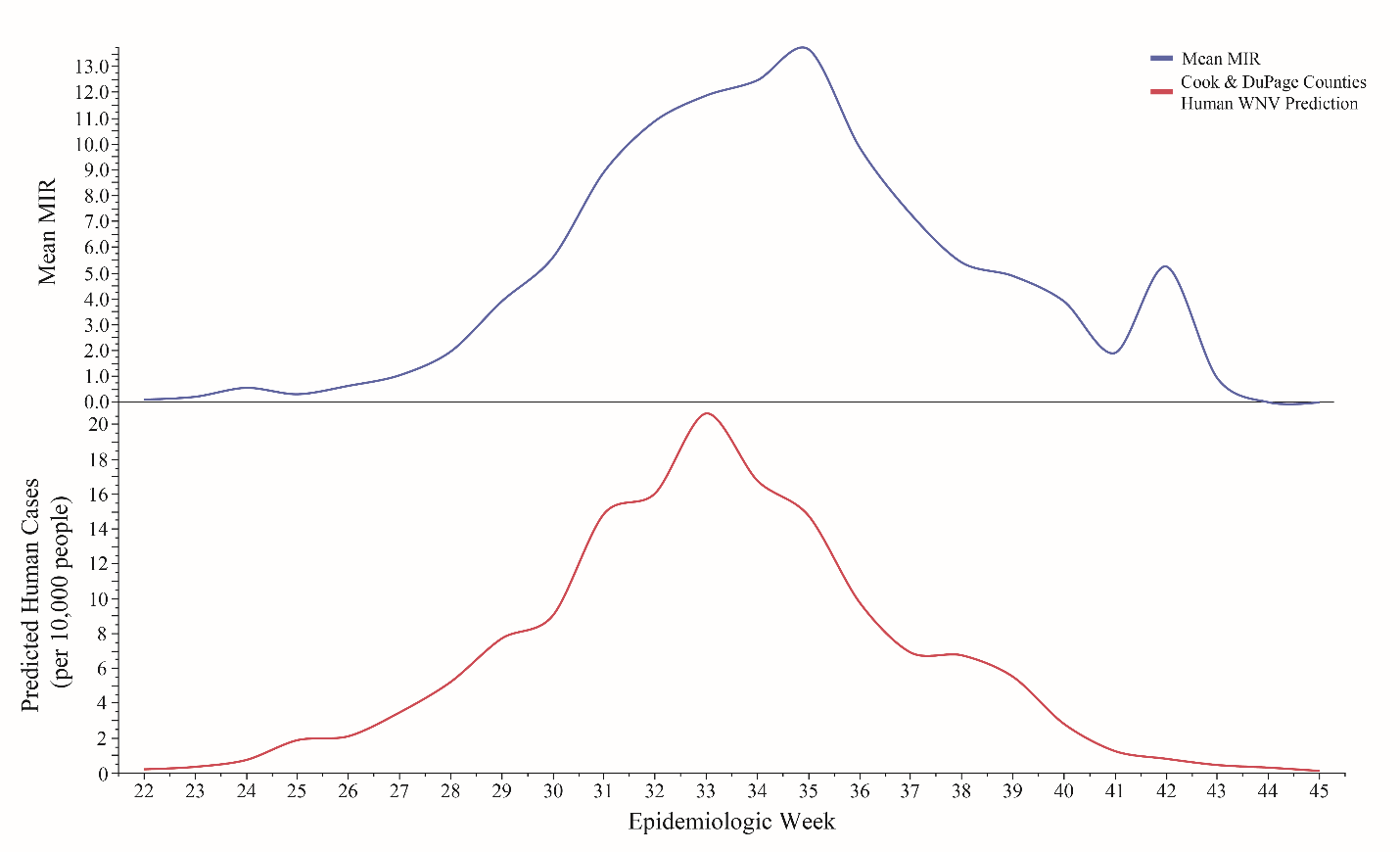
**Fig. S4.J.1.** Mean MIR and predicted human cases by epidemiological week (2012) for Cook and DuPage Counties.

*K. Model EAKF and L. Temperature Forced Model EAKF System*

Here, two forecasting systems are described, a baseline model with no temperature forcing [42] and a model that includes environmental forcing (average daily temperature [i.e., climatology] for the region) by accounting for temperature modulation of the extrinsic incubation period for mosquitos [43]. Both models used a standard susceptible-infected-recovered epidemiological construct and were optimized using a data assimilation method and two observed data streams: mosquito infection rates and reported human WNV cases. The forecast system relies on 3 components: (1) a core compartmental epidemiological model representing the transmission dynamics of WNV among mosquitoes and birds, as well as spillover to humans; (2) WNV surveillance data, i.e., vector mosquito WNV infection rates and reported human WNV cases; and (3) a data assimilation method (here, the ensemble adjustment Kalman filter [EAKF, 44]. The data assimilation method uses the surveillance data to recursively inform and optimize an ensemble of model simulations and, in so doing, provide an improved, posterior estimate of the true state as well as estimates of unobserved state variables and parameters. The forecasting is then generated in two successive steps. First, an ensemble of model simulations is recursively optimized using the ensemble adjustment Kalman filter and weekly observations of mosquito infection rates and human WNV cases until the week at which a forecast is to be initiated (in real time, this is the current week). Through the recursive ensemble adjustment Kalman filter optimization, model variables and parameters are better aligned with the local dynamics of the outbreak as thus far observed. Next, a forecast is generated by integrating the optimized ensemble of model simulations through to the end of the season.

In short, for each annual outbreak, a 300-member ensemble of the compartmental model was initiated (see S1 Text from DeFelice et al. 2018 for details on initial conditions and details on the model EAKF system) and run until the point of observation. Each week, available observations of human WNV cases and mosquito infection rates were assimilated into the model using the EAKF. Through this assimilation process, the model state space and parameters are iteratively updated to better represent current local outbreak dynamics. From week 20 until the end of the calendar year, starting with the first observation of infectious mosquitoes, forecasts were generated following the most recent update of the model state variables and parameters. That is, the forecasts were generated by integrating the latest posterior estimate for the WNV compartmental model [42,43] through time until the end of the outbreak season. This process was repeated weekly, with each successive forecast having one additional week of observational data assimilated. Each 300-member ensemble forecast was repeated 10 times with different randomly selected initial conditions and evaluated for accuracy according to prescribed forecast metrics (see DeFelice et al. 2018 for more details). The model requires active mosquito surveillance, human case data by week, at least one positive mosquito, estimated WNV prevalence by week, and at least 300 mosquito samples per week.

The accuracy of the forecasts was assessed through comparison of the forecast ensemble mean trajectory and observed outcomes. A short-term forecast was deemed accurate if the mean trajectory of human cases over the next 1, 2, 3, or 4 weeks was within ±25% or ±1 case of the total number cases, whichever was greater, during that time period. A seasonal forecast was deemed accurate if: 1) it peaked within ±1 week of the observed peak of infectious mosquitoes; 2) the maximum mosquito infection rate was within ±25% of the observed peak infection rate; 3) the total number of infectious mosquitoes over the entire season was within ±25% of the observed; and 4) the total number of human cases over the entire season was within ±25% or ±1 case of the total number of reported cases, whichever was larger. Additionally, forecasts were examined across all counties and years. The fraction of accurate forecasts was quantified for all forecasts grouped by the same lead time or by the same week of the year.

Seasonal forecast accuracy was also compared to historical average outbreaks to determine if the system could simply forecast accurately whether an outbreak was earlier or later than average or larger or smaller than average. The average outbreak for each county was defined as the mean value for the 4 metrics (total human WNV cases, total infectious mosquitoes, peak infectious mosquitoes and peak timing) for all years excluding the forecast year. A seasonal forecast was deemed accurate if the forecast coincided with the outbreak being either earlier or later than average or higher or lower than average. The performances of the temperature-forced and baseline models were compared using a Wilcoxon signed-rank test to assess whether forecast error of the two modeling approaches is statistically different. Absolute error was calculated and compared for predictions of observed peak of infectious mosquitoes, maximum mosquito infection rate, and the total number of human cases over the entire season, while root mean squared error (RMSE) was used to calculate the total weekly forecast error associated with the number of infectious mosquito observations over the season. For retrospective results see DeFelice et al. [43] and real-time results see DeFelice et al. [45].


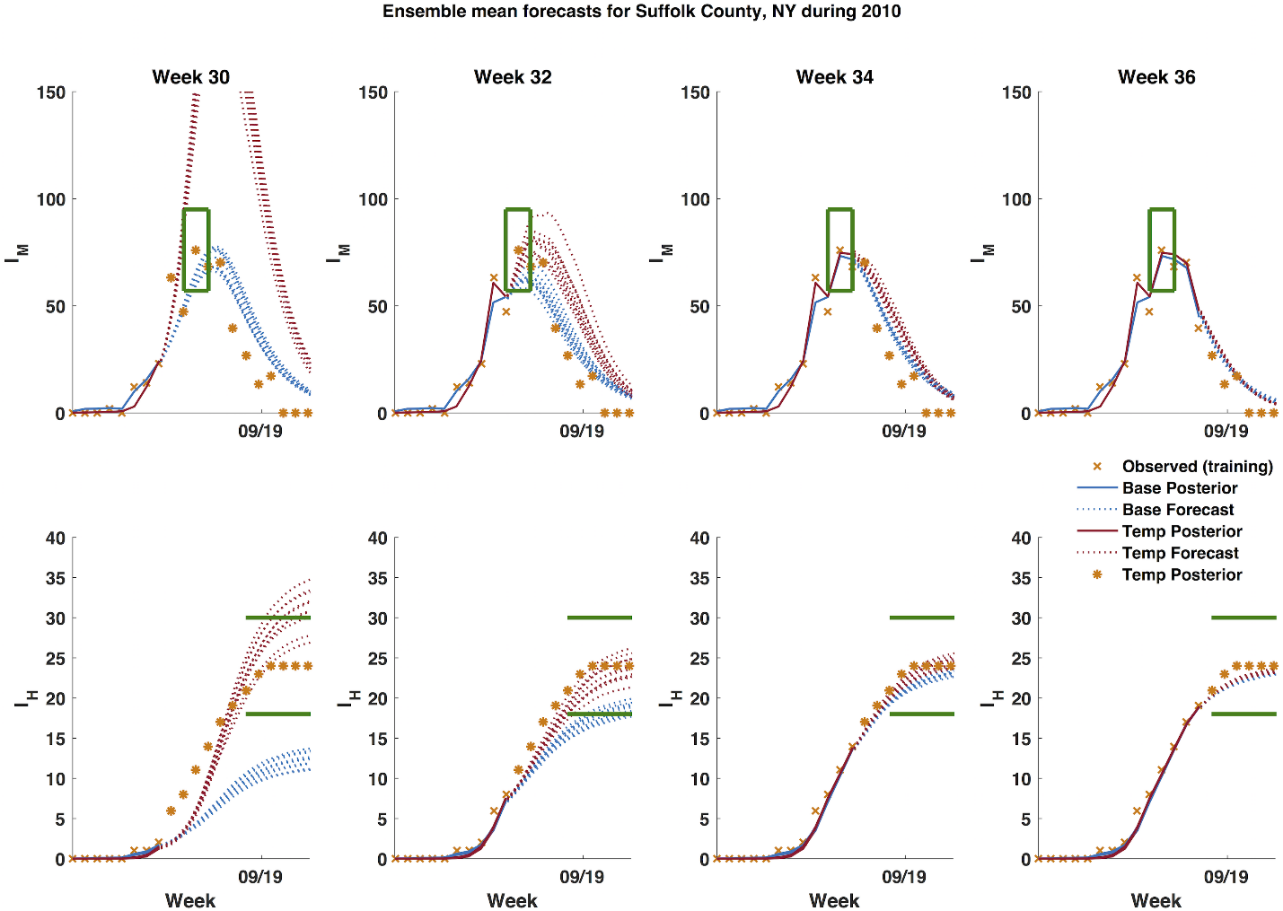


**Fig. S4.L.1.** Example forecasts of infectious mosquitoes and human WNV cases for Suffolk County, NY during 2010. Blue represents the baseline model and red represents the temperature-forced model. The dotted lines are the ensemble mean forecasts and solid lines are the ensemble mean posterior distribution, orange *x*’s are data points assimilated into the model, orange * are future observations, and the green lines represent the target range of an accurate forecast. A forecast was deemed accurate if: 1) peak timing was within ±1 week of the observed peak of infectious mosquitoes; 2) peak infection rate was within ±25% of the observed peak infection rate; and 3) human WNV cases were within ±25% of the total number of reported cases. Figure from [43] used under CC BY 4.0 license (https://creativecommons.org/licenses/by/4.0/).

*M. California Risk Assessment*

The California Mosquito-Borne Virus Surveillance and Response Plan estimates the risk of arbovirus transmission to humans using a flexible, semi-quantitative framework that can be applied across a wide variety of geographic scales and surveillance schemes [46]. The overall level of WNV risk is estimated as the average of all available risk elements 1) average daily temperature (“Environment”), 2) relative abundance of adult *Culex* mosquitoes versus the historical average (“Abundance”), 3) WNV infection prevalence in *Culex* mosquitoes (“Infection”), 4) sentinel chicken seroconversions (“Seroconversion”), 5) WNV infections in dead birds (“Dead Bird”), and 6) human cases. Because human cases are affected by reporting lags and thus are unreliable indicators of real-time risk, they are typically omitted from risk calculations that guide mosquito control operations during the season. Each surveillance element is assigned a value on an ordinal scale (1-5 for lowest to highest risk), and the mean value of all factors is calculated to estimate the WNV transmission risk and corresponding response level (i.e., normal season (1.0-2.5), emergency planning (2.6-4.0), and epidemic (4.1-5.0)). For the mosquito abundance factor, abundance is compared to the five-year average for the same area and time period. Viral infection rates are expressed as either minimum infection rates [MIR, 47] or maximum-likelihood estimates [MLE, 48] per 1,000 female mosquitoes tested. Due to differences in the attractiveness of traps to different subsets of the population, abundance and infection prevalence data are not pooled across trap types, but the most sensitive trap type’s value is used in the risk assessment. Also, due to differences in the sensitivity of traps between species and spatial heterogeneity in the distribution of *Cx. tarsalis* and *Cx. pipiens* complex mosquitoes relative to humans, separate risk calculations for each species are suggested.

Spatial and temporal scales are flexible for risk calculations depending on surveillance data availability for each factor (Fig S4.M.1). The use of control-relevant spatial units smaller than an entire agency, like city, zip code or operational zone, is preferred to capture spatial variation in mosquito and virus dynamics and define actionable targets for implementation of mosquito control, but surveillance data for at least one factor may not be available for these spatial scales. A biweekly timescale is commonly applied for risk calculations, but is flexible to range from weekly to monthly depending on surveillance data availability.

The risk assessment model has been widely used by mosquito abatement districts in California for the past two decades and more recently in other states such as New Jersey, with state-level customization of risk thresholds. Use of the risk model is aided by its implementation as interactive maps and an online calculator in the online data system, VectorSurv [49]. Statewide maps of risk surfaces are generated at 1/32° (~3.5-km) resolution weekly through the VectorSurv Gateway (Fig S4.M.1A-B) and are available to authenticated users as interactive maps for all current and historical time periods via the VectorSurv Maps website [50].


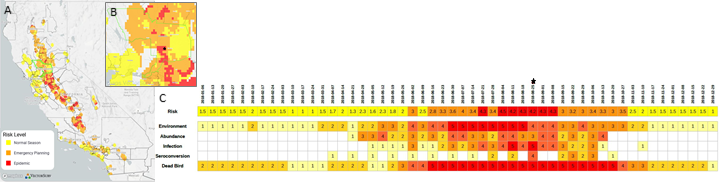
**Fig. S4.M.1**. Spatial and temporal estimates of West Nile virus risk from California Risk Assessment Model for 2018. Risk estimates spatially across A) California and B) Sacramento-Yolo Mosquito and Vector Control District (boundary outlined in green) for the week ending on Aug 25, 2018 (week starred in C). Vector control agency boundaries indicated in grey. Areas without risk values lack surveillance observation within 8 km. C) Risk estimates by week from Sacramento-Yolo Mosquito and Vector Control District in 2018 (outlined area in A & B). Abundance and infection in risk estimates for *Culex tarsalis* populations. Spatial maps from VectorSurv Maps website [50]. See text and [51] for risk estimation methodology.

**References**

1. Gorris, M.E., 2019. Ch 4: Climate controls on the spatial pattern of West Nile virus incidence in the United States. In *Environmental infectious disease dynamics in relation to climate and climate change*. University of California, Irvine. https://www.proquest.com/openview/4879510a3806015bd7fc27384b18dd18/1/advanced

2. Breiman L. Random forests. Machine learning. 2001;45: 5–32.

3. Daly C, Halbleib M, Smith JI, Gibson WP, Doggett MK, Taylor GH, et al. Physiographically sensitive mapping of climatological temperature and precipitation across the conterminous United States. International Journal of Climatology: a Journal of the Royal Meteorological Society. 2008;28: 2031–2064.

4. PRISM Climate Group. Parameter-elevation Regression on Independent Slopes Model. Oregon State University. http://prism.oregonstate.edu. 2019 [cited 28 Aug 2019]. Available: http://prism.oregonstate.edu

5. US Census Bureau. Intercensal estimates of the resident population for counties and states: April 1, 2000 to July 1, 2010. Suitland, MD: US Census Bureau. Retreived from: https://www.census.gov/data/datasets/time-series/demo/popest/intercensal-2000-2010-counties.html. 2017.

6. US Census Bureau. Population, Population Change, and Estimated Components of Population Change: April 1, 2010 to July 1, 2019 (CO-EST2019-alldata). Suitland, MD: US Census Bureau. Retreived from: https://www.census.gov/data/tables/time-series/demo/popest/2010s-counties-total.html. 2018.

7. Liaw A, Wiener M. Classification and Regression by randomForest. R News. 2002;2: 18–22.

8. R Core Team. R: A Language and Environment for Statistical Computing. Vienna, Austria: R Foundation for Statistical Computing; 2017. Available: https://www.R-project.org/

9. Shocket MS, Verwillow AB, Numazu MG, Slamani H, Cohen JM, El Moustaid F, et al. Transmission of West Nile and five other temperate mosquito-borne viruses peaks at temperatures between 23°C and 26°C. Franco E, Malagón T, Gehman A, editors. eLife. 2020;9: e58511. doi:10.7554/eLife.58511

10. Ryan SJ, McNally A, Johnson LR, Mordecai EA, Ben-Horin T, Paaijmans K, et al. Mapping Physiological Suitability Limits for Malaria in Africa Under Climate Change. Vector-Borne and Zoonotic Diseases. 2015;15: 718–725. doi:10.1089/vbz.2015.1822

11. Hess A, Davis JK, Wimberly MC. Identifying Environmental Risk Factors and Mapping the Distribution of West Nile Virus in an Endemic Region of North America. GeoHealth. 2018;2: 395–409. doi:10.1029/2018GH000161

12. Chuang T-W, Hockett CW, Kightlinger L, Wimberly MC. Landscape-level spatial patterns of West Nile virus risk in the northern Great Plains. The American Journal of Tropical Medicine and Hygiene. 2012;86: 724–731.

13. Wimberly MC, Giacomo P, Kightlinger L, Hildreth MB. Spatio-Temporal Epidemiology of Human West Nile Virus Disease in South Dakota. International Journal of Environmental Research and Public Health. 2013;10: 5584–5602. doi:10.3390/ijerph10115584

14. Keyel AC, Elison Timm O, Backenson PB, Prussing C, Quinones S, McDonough KA, et al. Seasonal temperatures and hydrological conditions improve the prediction of West Nile virus infection rates in Culex mosquitoes and human case counts in New York and Connecticut. PLOS ONE. 2019;14: e0217854. doi:10.1371/journal.pone.0217854

15. Manson S, Schroeder J, Van Riper D, Ruggles S. IPUMS National Historical Geographic Information System: Version 12.0 [Database]. 2017. Available: ttp://doi.org/10.18128/D050.V12.0

16. Homer C, Dewitz J, Yang L, Jin S, Danielson P, Xian G, et al. Completion of the 2011 National Land Cover Database for the conterminous United States–representing a decade of land cover change information. Photogrammetric Engineering & Remote Sensing. 2015;81: 345–354.

17. Xia Y, Mitchell K, Ek M, Cosgrove B, Sheffield J, Luo L, et al. Continental‐scale water and energy flux analysis and validation for North American Land Data Assimilation System project phase 2 (NLDAS‐2): 2. Validation of model‐simulated streamflow. Journal of Geophysical Research: Atmospheres. 2012;117.

18. Lawler JJ, Edwards Jr TC. A variance-decomposition approach to investigating multiscale habitat associations. The Condor. 2006;108: 47–58.

19. Williams CJ, Moffitt CM. Estimation of pathogen prevalence in pooled samples using maximum likelihood methods and open-source software. Journal of Aquatic Animal Health. 2005;17: 386–391.

20. Meinshausen N. Quantile regression forests. Journal of Machine Learning Research. 2006;7: 983–999.

21. Meinshausen N. quantregForest: Quantile Regression Forests. 2017. Available: https://CRAN.R-project.org/package=quantregForest

22. Little E, Campbell SR, Shaman J. Development and validation of a climate-based ensemble prediction model for West Nile Virus infection rates in *Culex* mosquitoes, Suffolk County, New York. Parasit Vectors. 2016;9: 443.

23. Smith KH, Tyre AJ, Hamik J, Hayes MJ, Zhou Y, Dai L. Using Climate to Explain and Predict West Nile Virus Risk in Nebraska. GeoHealth. 2020;4: e2020GH000244. doi:10.1029/2020GH000244

24. Wood SN. Fast stable restricted maximum likelihood and marginal likelihood estimation of semiparametric generalized linear models. Journal of the Royal Statistical Society (B). 2011;73: 3–36.

25. Abatzoglou JT, McEvoy DJ, Redmond KT. The West Wide Drought Tracker: drought monitoring at fine spatial scales. Bulletin of the American Meteorological Society. 2017;98: 1815–1820.

26. Vose RS, Applequist S, Squires M, Durre I, Menne MJ, Williams CN, et al. NOAA’s Climate Divisional Database (nCLIMDIV). National Climatic Data Center; 2014. Available: https://doi.org/10.7289/V5M32STR

27. Akaike H. A new look at the statistical model identification. IEEE Transactions on Automatic Control. 1974;19: 716–723. doi:10.1109/TAC.1974.1100705

28. Burnham KP, Anderson DR. Model selection and Multimodel inference. New York: Springer-Verlag; 2002.

29. Paull SH, Horton DE, Ashfaq M, Rastogi D, Kramer LD, Diffenbaugh NS, et al. Drought and immunity determine the intensity of West Nile virus epidemics and climate change impacts. Proc R Soc B. 2017;284: 20162078.

30. Mitchell KE, Lohmann D, Houser PR, Wood EF, Schaake JC, Robock A, et al. The multi‐institution North American Land Data Assimilation System (NLDAS): Utilizing multiple GCIP products and partners in a continental distributed hydrological modeling system. Journal of Geophysical Research: Atmospheres. 2004;109: 1–32.

31. Lachenbruch PA, Mickey MR. Estimation of Error Rates in Discriminant Analysis. Technometrics. 1968;10: 1–11. doi:10.2307/1266219

32. Poh KC, Chaves LF, Reyna-Nava M, Roberts CM, Fredregill C, Bueno R Jr, et al. The influence of weather and weather variability on mosquito abundance and infection with West Nile virus in Harris County, Texas, USA. Sci Total Environ. 2019;675: 260–272. doi:10.1016/j.scitotenv.2019.04.109

33. Farrington C. Estimating prevalence by group testing using generalized linear models. Statistics in medicine. 1992;11: 1591–1597.

34. Shumway R, Stoffer D. Time series analysis and its applications. 3rd ed. New York: Springer; 2011.

35. Didan K. MOD13Q1: MODIS/Terra Vegetation Indices 16-Day L3 Global 250 m SIN Grid V006. http://dx.doi.org/10.5067/MODIS/MOD13Q1.006. 2015.

36. Chaves LF, Pascual M. Comparing Models for Early Warning Systems of Neglected Tropical Diseases. PLOS Neglected Tropical Diseases. 2007;1: 1–6. doi:10.1371/journal.pntd.0000033

37. Davis JK, Vincent G, Hildreth MB, Kightlinger L, Carlson C, Wimberly MC. Integrating Environmental monitoring and mosquito surveillance to predict vector-borne disease: prospective forecasts of a West Nile virus outbreak. PLoS Currents. 2017;9.

38. Davis JK, Vincent GP, Hildreth MB, Kightlinger L, Carlson C, Wimberly MC. Improving the prediction of arbovirus outbreaks: A comparison of climate-driven models for West Nile virus in an endemic region of the United States. Acta Trop. 2018;185: 242–250.

39. Abatzoglou JT. Development of gridded surface meteorological data for ecological applications and modelling. International Journal of Climatology. 2013;33: 121–131.

40. Gorelick N, Hancher M, Dixon M, Ilyushchenko S, Thau D, Moore R. Google Earth Engine: Planetary-scale geospatial analysis for everyone. Remote Sensing of Environment. 2017. doi:10.1016/j.rse.2017.06.031

41. Karki S, Brown WM, Uelmen J, Ruiz MO, Smith RL. The drivers of West Nile virus human illness in the Chicago, Illinois, USA area: Fine scale dynamic effects of weather, mosquito infection, social, and biological conditions. PLOS ONE. 2020;15: e0227160. doi:10.1371/journal.pone.0227160

42. DeFelice NB, Little E, Campbell SR, Shaman J. Ensemble forecast of human West Nile virus cases and mosquito infection rates. Nature Communications. 2017;8: 14592.

43. DeFelice NB, Schneider ZD, Little E, Barker C, Caillouet KA, Campbell SR, et al. Use of temperature to improve West Nile virus forecasts. PLOS Comput Biol. 2018;14. doi:10.1371/journal.pcbi.1006047

44. Anderson JL. An ensemble adjustment Kalman filter for data assimilation. Monthly Weather Review. 2001;129: 2884–2903.

45. DeFelice NB, Birger R, DeFelice N, Gagner A, Campbell SR, Romano C, et al. Modeling and Surveillance of Reporting Delays of Mosquitoes and Humans Infected With West Nile Virus and Associations With Accuracy of West Nile Virus Forecasts. JAMA Network Open. 2019;2: e193175–e193175. doi:10.1001/jamanetworkopen.2019.3175

46. CDPH. California Department of Public Health, Mosquito and Vector Control Association of California, and University of California, California mosquito-borne virus surveillance and response plan. 2020. Available: https://westnile.ca.gov/download.php?download_id=4502

47. Reeves WC, Hammon WM. The role of arthropod vectors. Epidemiology of the arthropod-borne viral encephalitides in Kern County, California, 1943-1952. Berkeley, CA: University of California Press; 1962. pp. 75–190.

48. Hepworth G, Biggerstaff BJ. Bias correction in estimating proportions by pooled testing. Journal of Agricultural, Biological and Environmental Statistics. 2017;22: 602–614.

49. VectorSurv Development Team. VectorSurv: Vectorborne Disease Surveillance System. 2020. Available: https://vectorsurv.org

50. VectorSurv Development Team. VectorSurv Maps. 2020. Available: https://maps.vectorsurv.org

51. VectorSurv Development Team. Risk assessment surface methodology. 2020. Available: https://vectorsurv.org/
